# ADAMTS18^+^ villus tip telocytes maintain a polarized VEGFA signaling domain and fenestrations in nutrient-absorbing intestinal blood vessels

**DOI:** 10.1038/s41467-022-31571-2

**Published:** 2022-07-09

**Authors:** Jeremiah Bernier-Latmani, Cristina Mauri, Rachel Marcone, François Renevey, Stephan Durot, Liqun He, Michael Vanlandewijck, Catherine Maclachlan, Suzel Davanture, Nicola Zamboni, Graham W. Knott, Sanjiv A. Luther, Christer Betsholtz, Mauro Delorenzi, Cathrin Brisken, Tatiana V. Petrova

**Affiliations:** 1 Department of Oncology, Ludwig Institute for Cancer Research Lausanne and University of Lausanne, Lausanne, Switzerland; 2 Bioinformatics Core Facility, SIB Swiss Institute of Bioinformatics, Lausanne, Switzerland; 3 Department of Immunobiology, University of Lausanne, Lausanne, Switzerland; 4 Institute of Molecular Systems Biology ETH, Zurich, Switzerland; 5 Department of Immunology, Genetics and Pathology, Rudbeck Laboratory, Uppsala University, Uppsala, Sweden; 6 Department of Medicine-Huddinge, Karolinska Institutet, Huddinge, Sweden; 7 Bio Electron Microscopy Laboratory, School of Life Sciences, EPFL, Lausanne, Switzerland; 8 Swiss Institute for Experimental Cancer Research (ISREC), School of Life Sciences, EPFL, Lausanne, Switzerland

**Keywords:** Body patterning, Angiogenesis, Metabolomics

## Abstract

The small intestinal villus tip is the first point of contact for lumen-derived substances including nutrients and microbial products. Electron microscopy studies from the early 1970s uncovered unusual spatial organization of small intestinal villus tip blood vessels: their exterior, epithelial-facing side is fenestrated, while the side facing the villus stroma is non-fenestrated, covered by pericytes and harbors endothelial nuclei. Such organization optimizes the absorption process, however the molecular mechanisms maintaining this highly specialized structure remain unclear. Here we report that perivascular LGR5^+^ villus tip telocytes (VTTs) are necessary for maintenance of villus tip endothelial cell polarization and fenestration by sequestering VEGFA signaling. Mechanistically, unique VTT expression of the protease ADAMTS18 is necessary for VEGFA signaling sequestration through limiting fibronectin accumulation. Therefore, we propose a model in which LGR5^+^ ADAMTS18^+^ telocytes are necessary to maintain a “just-right” level and location of VEGFA signaling in intestinal villus blood vasculature to ensure on one hand the presence of sufficient endothelial fenestrae, while avoiding excessive leakiness of the vessels and destabilization of villus tip epithelial structures.

## Abstract (web summary)

The molecular mechanisms ensuring the specialized structure of small intestinal villus tip blood vessels are incompletely understood. Here the authors show that ADAMTS18^+^ telocytes maintain a “just-right” level and location of VEGFA signaling on intestinal villus blood vessels, thereby ensuring the presence of endothelial fenestrae for nutrient absorption, while avoiding excessive leakiness and destabilization of villus tip epithelial structures.

## Introduction

VEGFA is a potent hypoxia-driven angiogenic factor essential for developmental and pathological angiogenesis^1^. In adult animals VEGFA-dependent vascular beds are limited to organs where rapid solute exchange is necessary for proper function including the kidney, pancreas, endocrine organs and the small intestine^2,3^. Small intestinal villi harbor distinct blood vessels specialized for nutrient uptake, enabling absorption rates at least 100-fold higher than other vessels^4^. VEGFA is highly expressed in intestinal villi and this expression is driven by a gradient of decreasing pO_2_ towards the villus tip^5^. It has been proposed that this hypoxic gradient is created by the hairpin turn in blood flow at the villus tip which creates a countercurrent exchange whereby O_2_ diffusing from arterioles is shunted to venules before reaching the villus tip^6^.

VEGFA signaling increases proliferation, migration, survival, sprouting and permeability of endothelial cells^1^. Correspondingly, VEGFA maintains a dense, filopodia-rich adult intestinal villus vasculature^2,3,7,8^. VEGFA signaling is highlighted at the villus tip by endothelial cell expression of VEGFR3 and ESM1^7,9^, markers of active VEGFA signaling during development and in other adult vessel beds^3,10–14^. VEGFA also promotes blood vessel permeability through the formation of small pores in the endothelial cell membrane, called fenestrations^2,3,15–17^. Endothelial fenestrations are highly dependent on VEGFA signaling as blockade of this pathway restores a non-fenestrated endothelial membrane^2,3^. In the small intestine, most nutrient absorption occurs at the villus tip^18,19^ and electron microscopy (EM) revealed that villus tip vessels maintain a fenestrated endothelium^20,21^, likely accounting for their efficient nutrient absorption capacity. Intriguingly, fenestrations are restricted to the endothelial membrane facing the intestinal epithelium, while endothelial nuclei are positioned towards the villus core^22,23^. This polarization ensures an absorptive endothelial surface directed towards epithelial cells, the source of nutrient entry, however, mechanisms maintaining this unique cellular architecture remain unknown. Here, we show that a population of telocytes maintains villus tip endothelial cell polarization by restricting VEGFA accumulation through ADAMTS18-dependent extracellular matrix proteolysis.

## Results

### Villus tip endothelial cell polarization is VEGFA-dependent

**Fig. 1:**
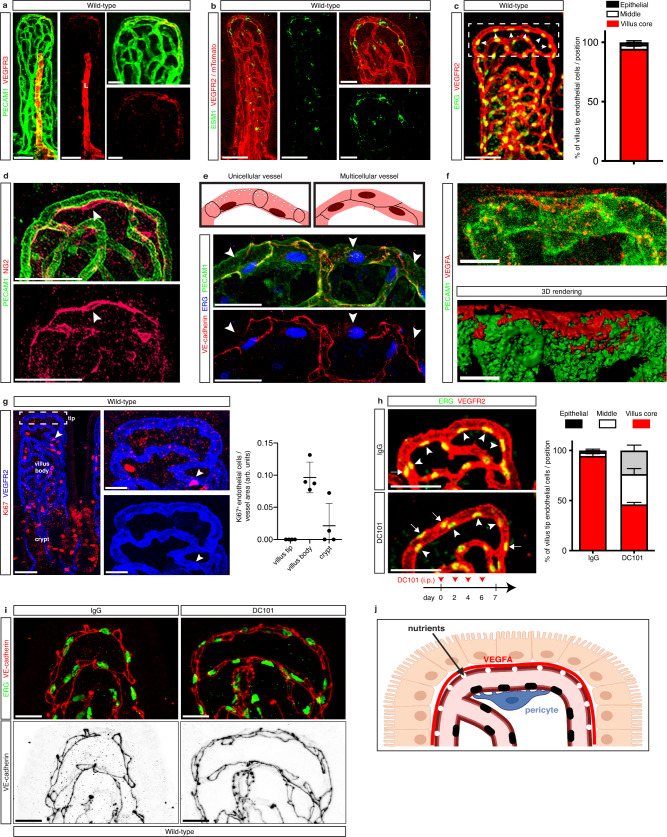
Intestinal villus tip endothelial cell polarization is VEGFA-dependent. **a**, **b** Villus tip endothelial cells display high VEGFA signaling. Staining for (**a**) VEGFR3 (red) and PECAM1 (green) and (**b**) ESM1 (green) and VEGFR2 (red) of adult (**a**) C57BL/6 and (**b**) mTomato small intestinal villus tip vessels. L, lymphatic vessel. **c** Endothelial cell nuclei are polarized to the villus core side of villus tip vessels. Staining for ERG (green) and VEGFR2 (red) in C57BL/6 adult mice. Box denotes villus tip area. Quantification of endothelial cell nuclei positioning in the villus tip vessel; *n* = 3 mice. **d** Pericytes are positioned on the villus core side of villus tip vessels. Staining for NG2 (red, arrowhead) and PECAM1 (green). **e** Villus tip vessels consist of unicellular endothelial tubes. Cartoon representing differences in cell-cell junction patterning (black lines) between multicellular and unicellular vessels. Villus tip vessels (PECAM1, green) display lumenized endothelial cells based on cell-cell junction immunostaining (VE-cadherin, red) and “open faces” (arrowheads) towards epithelial cells. **f** VEGFA is concentrated to the epithelial side of villus tip vessels. Staining for VEGFA (red) and PECAM1 (green); bottom, 3D rendering of top image. **g** Villus tip endothelial cells do not proliferate. Staining for VEGFR2 (blue) and Ki67 (red) in C57BL/6 adult mice. Quantification of Ki67^+^ endothelial cells in the crypts, villus body (arrowhead) and villus tips; *n* = 4 mice. **h** Villus tip endothelial cell nuclei polarization is VEGFA-dependent. Staining for VEGFR2 (red) and ERG (green). Quantification of endothelial cell nuclei position on the villus core (arrowheads), middle, or epithelial sides (arrows) of the villus tip vessel in control- and DC101-treated adult C57BL/6 mice; *n* = 3 mice. **i** Unicellular arrangement of villus tip blood vessels is VEGFA-dependent. Staining for endothelial cell nuclei (green, ERG) and endothelial cell junctions (red, VE-cadherin). **j** Villus tip endothelial nuclei are positioned on the villus core side of the vessel, aligning with pericytes, while the endothelial membrane with fenestrations faces the epithelium. VEGFA is sequestered on the epithelium-facing part of the vessel (created with biorender.com). All microscopic images were generated by whole-mount immunostaining. Scale bars: 50 μm: **a**, **b**, **c**, **d**, **g**, **h**; 20 μm: **e**, **g** (inset), **i**; 10 μm: **f**. All values shown as mean ± SD. Source data are provided as a Source Data file.

To confirm previous EM observations of the small intestinal villus tip, we analyzed this structure by whole-mount immunostaining. As expected, markers of high endothelial VEGFA signaling VEGFR3 and ESM1^3,10–14^ were enriched in villus tip vessels (Fig. 1a, b; Supplementary Fig. 1a), corresponding with high villus tip hypoxia (Supplementary Fig. 1b) and their expression was decreased after VEGFA signaling blockade (Supplementary Fig. 1c). We confirmed that endothelial cell nuclei at the villus tip are polarized to the villus core side of the vessel and are absent from the epithelial side by staining for the endothelial transcription factor ERG (Fig. 1c). Typically, a single pericyte was detected on the villus core side of the villus tip endothelial cells as determined by staining for NG2 (Fig. 1d). In normal vessels, endothelial cells are evenly spaced so that any given vessel segment is composed of at least two endothelial cells. During development, blood vessel anastomosis and regression impose distinct endothelial cell arrangements to maintain vessel patency. Some of these cellular arrangements result in single lumenized endothelial cells which can be recognized by staining for endothelial cell junctions^24^. Staining for the endothelial adherens junction protein VE-cadherin revealed most villus tip endothelial cells take on this unicellular tube configuration (Fig. 1e). This arrangement allows the epithelial side of endothelial cells to be devoid of a nucleus and cell junctions, instead it can present an “open-faced” fenestrated membrane for nutrient absorption.

Cell membrane fenestration is limited to the epithelial side of villus tip endothelial cells^22^ and VEGFA is a well-known inducer of fenestration^2,3,15–17^. Given this particular distribution we hypothesized that VEGFA was sequestered to the epithelial side of villus tip endothelial cells. Immunostaining revealed that VEGFA was preferentially deposited on the epithelial side of villus tip endothelial cells, consistent with our hypothesis (Fig. 1f).

One main outcome of VEGFA signaling is increased endothelial cell proliferation^1^. Given the high level of VEGFA signaling at the villus tip we analyzed endothelial cell proliferation by immunostaining for Ki67. Spatial analysis showed rare Ki67^+^ endothelial cells limited to the villus body, while no Ki67^+^ endothelial cells were observed at the villus tip (Fig. 1g). These observations suggest that VEGFA signaling at the villus tip does not promote proliferation, rather is necessary to maintain a fenestrated endothelium poised for nutrient absorption.

We next tested whether VEGFA signaling regulates villus tip endothelial cell polarization by treating mice with the antibody DC101, which blocks the interaction of VEGFA with its receptor VEGFR2^25^. DC101-treated mice displayed markedly randomized distribution of villus tip endothelial nuclei, which were found equally on the epithelial and villus core side of the vessel (Fig. 1h). Additionally, analysis of endothelial cell junctions revealed a switch from unicellular tubes to multicellular vessels at the villus tip (Fig. 1i). These results show that sequestered VEGFA signaling at the villus tip is necessary for maintenance of the polarized endothelial cell phenotype (Fig. 1j).

### *Lgr5*^+^ villus tip telocytes are aligned with polarized villus tip vessels

Since VEGFA signaling maintained distinct endothelial patterning at the villus tip we sought to identify cells controlling VEGFA availability. VEGFA is sequestered to the epithelial side of endothelial cells (Fig. 1f). Stromal cells are positioned in the space between epithelial cells and blood vessels and form a sheath around the intestinal villus core^26,27^. These cells are alternatively labeled as subepithelial fibroblasts, myofibroblasts^28^ or telocytes^27^ and we hypothesized they could play a role in controlling VEGFA availability at the villus tip.

**Fig. 2:**
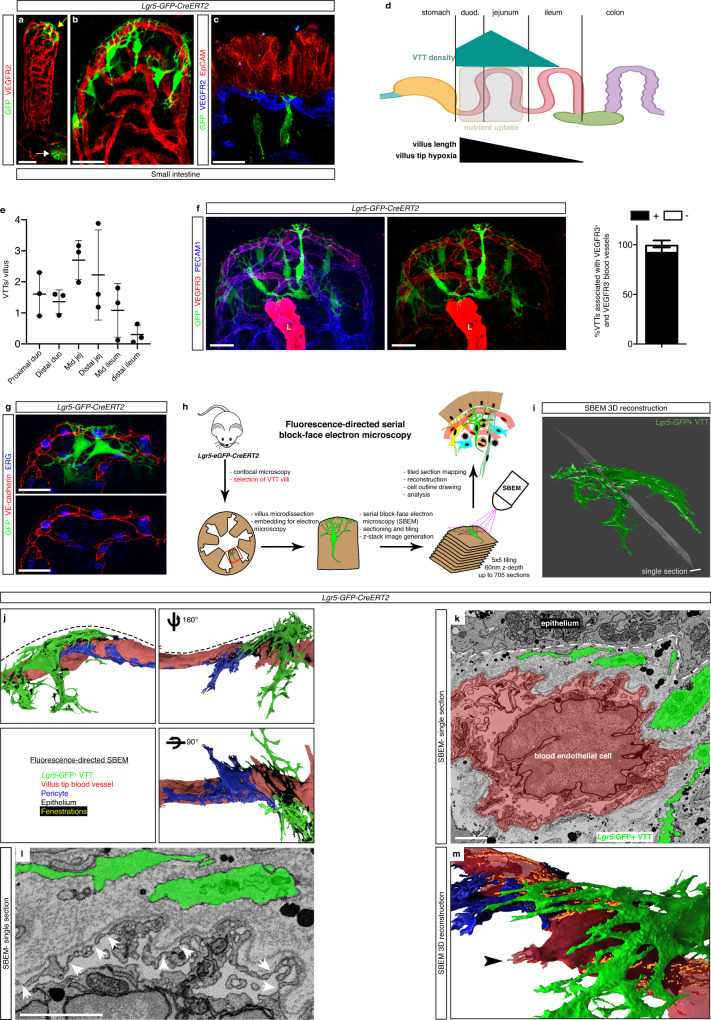
*Lgr5*^+^ villus tip telocytes are aligned with polarized villus tip vessels. **a**, **b** *Lgr5*^+^-GFP villus tip telocytes (VTTs, yellow arrow) are distinct from *Lgr5*^+^ epithelial stem cells (white arrow) and are associated with villus tip blood vessels. Staining for VEGFR2 (red) and GFP (green). **c** VTT extensions are located between villus tip vessels and epithelial cells, the cell body is in the villus core. Staining for VEGFR2 (blue), GFP (green) and EpCAM (red). **d** Scheme of relative VTT density along the small intestine. VTTs are enriched in the proximal small intestine, corresponding to the zone with longest villus length and highest villus tip hypoxia (created with biorender.com). **e** Quantification of number of VTTs per villus in indicated intestinal regions; *n* = 3 mice. **f** VTTs are associated with high VEGFA signaling villus tip endothelial cells. Staining for PECAM1 (blue), VEGFR3 (red) and GFP (green). Quantification of percentage VTT associated with either VEGFR3^+^ or VEGFR3^−^ villus tip vessels of *Lgr5-GFP-CreERT2* mice; *n* = 3 mice. L, lymphatic vessel. **g** VTTs drape cellular extensions over villus tip endothelial cell “open-faces”. Staining for VE-cadherin (red), ERG (blue) and GFP (green). **h** Workflow of fluorescence-directed serial block-face electron microscopy (SBEM) in *Lgr5-EGFP-CreERT2* adult mice. **i** 3D reconstruction of a VTT derived from hand tracing across serial electron micrographs. **j** VTTs drape extensions between the villus tip vessel and the epithelium, while pericytes extend along the opposite villus core side of the vessel. 3D reconstruction of the villus tip from serial EM images of a *Lgr5-GFP-CreERT2* mouse. Pseudo-coloring for VTTs (green), blood vessels (red), pericyte (blue), fenestrations (yellow). Epithelium, dotted line. **k** VTTs are separated from villus tip endothelial cells by a fibrillar extracellular matrix (dotted line). Single-electron micrograph section from SBEM images in (**j**), pseudo-coloring for an endothelial cell (red) and a VTT (green). Epithelium, dotted line. **l**, **m** VTTs and endothelial fenestrations are aligned. **l** Single-electron micrograph, VTT (green), endothelial fenestrations (arrowheads). **m** Magnification of the 3D rendering from (**j**); fenestrations (orange), endothelial filopodium (arrowhead). Whole-mount immunostaining was used to generate images **a**–**c**, **f** and **g**; electron microscopy-generated images **i**–**m**. Scale bars: 50 μm: **a**, **b**; 20 μm: **c**, **f**, **g**; 2 μm: **k**, **l**. All values shown as mean ± SD. Source data are provided as a Source Data file.

Lacking a specific marker for villus tip subepithelial cells we serendipitously found one while analyzing *Lgr5-eGFP-CreERT2* mice^29^. These mice are a reporter for epithelial crypt ISCs (Fig. 2a), however whole-mount immunostaining revealed a second, villus tip stromal GFP^+^ cell population (Fig. 2a, b). These cells have a distinct elongated shape and their distal ends are composed of dense, highly branched processes wrapped around the villus tip blood capillary and positioned directly between villus tip epithelial and endothelial cells, while the main cell body with short processes extends into the villus core (Fig. 2b, c). Recently, *Lgr5*^+^ villus tip stromal cells interacting with epithelial cells were described as villus tip telocytes (VTTs)^30^; for clarity we will use the same nomenclature.

Perivascular cells in other organs include vascular smooth muscle cells, pericytes and nervous system cells^31^ and we previously observed these cell types to be in close proximity to villus blood capillaries^7,32^. Therefore, we performed wholemount immunostaining for markers of perivascular cells to determine if they were expressed in the perivascular VTTs. However, *Lgr5*^+^ VTTs were negative for markers of villus smooth muscle cells (αSMA), pericytes (NG2), and nervous system cells (PGP9.5, S100, GFAP) in *Lgr5-GFP-CreERT2* mice (Supplementary Fig. 2a-e). In all, these results suggest that VTTs are a novel perivascular cell type.

Most nutrient uptake occurs in proximal parts of intestine, such as the duodenum and upper jejunum^33^. Accordingly, villus size is largest in the duodenum, near the stomach, and it progressively decreases in the jejunum and ileum (Fig. 2d). To determine whether VTT abundance varied along the anterior-posterior intestinal axis, we quantified their number per villus in each zone. Highest numbers of *Lgr5*^+^ VTTs were observed in the duodenum and jejunum, while few VTTs were observed in the lower ileum (Fig. 2d, e). Of note, transgene expression in *Lgr5-GFP-CreERT2* mice is mosaic with ~30% of crypts harboring GFP^+^ cells, while 100% of ISCs express *Lgr5* mRNA^29,30^. Therefore, the number of *Lgr5*^+^ VTTs are likely underrepresented in this analysis. Nevertheless, these results show that *Lgr5*^+^ VTTs are enriched in the proximal, nutrient-absorbing part of small intestine.

Analysis of small intestinal villi in *Lgr5-GFP-CreERT2* pups revealed that although GFP^+^ ISCs are observed at birth, VTTs were not present. Rather, GFP^+^ VTTs were observed to accumulate progressively after birth, suggesting that VTTs emerge during the postnatal intestinal maturation process (Supplementary Fig. 3).

Villus tip endothelial cells express markers of high VEGFA signaling, including VEGFR3 and ESM1 (see Fig. 1a, b). Furthermore, we observed villus tip endothelial cells present an “open-face”, without cell junctions to their epithelial side (see Fig. 1e). To determine if VTTs were associated with villus tip endothelial cells with active VEGFA signaling we performed immunostaining for VEGFR3 and GFP in *Lgr5-GFP-CreERT2* mice. Near 100% of VTTs had cellular extensions draped over VEGFR3^+^ blood vessels (Fig. 2f). Furthermore, VTT extensions also covered the endothelial cell open face (Fig. 2g). In all, VTTs are aligned with mature, polarized villus tip endothelial cells.

To characterize villus tip endothelial cell-VTT organization at the ultrastructural level, we performed fluorescence-directed serial block-face scanning electron microscopy (SBEM)^34^, allowing 3D, nanometer-resolution reconstruction of GFP^+^ VTTs and nearby cells at the villus tip (Fig. 2h, i; Supplementary Fig. 4a; Supplementary Movie 1). As expected, VTTs were located in the subepithelial space and displayed a highly branched morphology both on the epithelial side of the vessel and in the villus core (Fig. 2i, j). Electron-dense particles resembling chylomicrons were observed between the web-like highly branched VTT processes (Fig. 2k). VTTs harbored euchromatic nuclei and their cytoplasm contained ample mitochondria, endoplasmic reticula and Golgi complexes, suggesting these cells are transcriptionally and metabolically active (Supplementary Fig. 4b).

VTTs were highly integrated into the subepithelial zone as quantification of their cellular interactome revealed that the majority (~70%) of direct cell contacts were either with other subepithelial fibroblasts/telocytes or with epithelial cells (Supplementary Fig. 4c-f). VTT processes also extended into the villus core and directly contacted immune cells, enteric glia and villus core fibroblasts (Supplementary Fig. 4e, f). Although VTTs were perivascular, they rarely were in direct contact with endothelial cells (Fig. 2j-m; Supplementary Fig. 4f), rather they were separated by a fibrillar extracellular matrix (ECM) distinct from the endothelial basement membrane (BM; Fig. 2k, l). In contrast, pericytes, located on the opposite side of vessels, made numerous direct cell contacts with endothelial cells (Fig. 2j; Supplementary Fig. 4g). Endothelial fenestrations were aligned with VTT processes along the epithelial side of villus tip vessels but were rare in pericyte-covered endothelium and absent in filopodia-displaying endothelial cells (Fig. 2l, m). Taken together, our results reveal VTTs as perivascular cells uniquely positioned at the villus tip to regulate VEGFA availability and hence villus tip vessel patterning.

### VTTs constrain villus tip VEGFA signaling

**Fig. 3:**
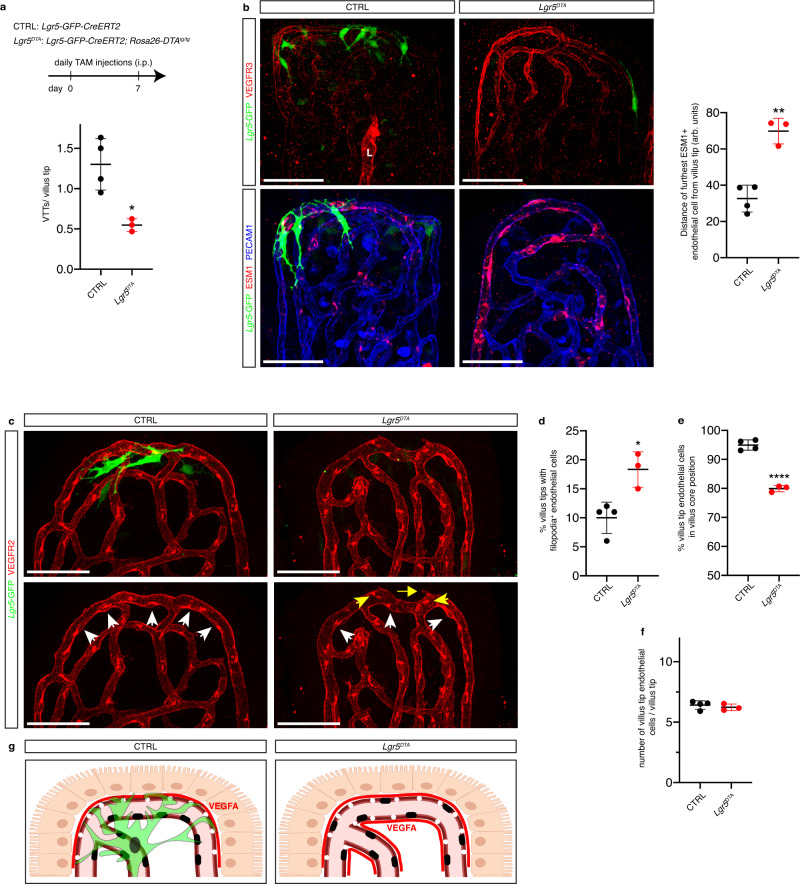
VTTs constrain villus tip VEGFA signaling. **a** Reduced number of VTTs in *Lgr5*^*DTA*^ mice. Quantification of VTTs/villus in control *Lgr5-GFP-CreERT2* and *Lgr5*^*DTA*^ mice after 1 week of deletion (*p* = 0.0112, *n* = 3–4 mice). **b** VTT depletion increases villus tip endothelial VEGFA signaling. Staining for PECAM1 (blue) and VEGFR3 (red, top) or ESM1 (red, bottom). L, lymphatic vessel. Quantification of distance at which ESM1^+^ vessels are observed from the villus tip in either control or *Lgr5*^*DTA*^ mice after 1 week of deletion (*p* = 0.0011, *n* = 3-4 mice). **c**–**f** VTT depletion disturbs villus tip vessel polarization and phenotype. **c** Endothelial cell nuclei are polarized towards the villus core (white arrowheads, perinuclear VEGFR2 staining) or epithelial side (yellow arrowheads) of villus tip vessels. Endothelial cells displaying filopodia (yellow arrows). Staining for VEGFR2 (red) and GFP (green). **d**–**f** Quantification of the (**d**) percentage of villus tip endothelial cells displaying filopodia (*p* = 0.0123), (**e**) percentage of endothelial cells on the villus core side of the vessel (*p* < 0.0001) and (**f**) the number of endothelial cells/villus tip in control and *Lgr5*^*DTA*^ mice; *n* = 3–4 mice. **g** Scheme of VEGFA signaling phenotype in the presence (left) or absence (right) of VTTs. The presence of VTTs confines VEGFA signaling to villus tip vessels and promotes villus tip endothelial cell nucleus polarization. VTT depletion increases VEGFA signaling and disturbs villus tip endothelial cell polarization (created with biorender.com). All microscopic images were generated from whole-mount immunostaining. Scale bars: 50 μm. *P < 0.05, **P < 0.01, ****P < 0.0001 2-tailed unpaired Student’s *t* test. All values shown as mean ± SD. Source data are provided as a Source Data file.

To study the function of *Lgr5*^+^ VTTs we generated *Lgr5-GFP-CreERT2; Rosa26-DTA* mice (*Lgr5*^*DTA*^), which express diphtheria toxin A under the control of the *Lgr5* promoter. ISC DTA expression does not induce crypt or intestinal epithelial cell defects^35–37^, however, tamoxifen injection resulted in a significant reduction of jejunal *Lgr5*^+^ VTTs (Fig. 3a). We investigated the impact of VTT depletion on VEGFA signaling in villus tip endothelial cells by staining for ESM1 and VEGFR3. The number of VEGFR3- and ESM1-positive villus tip endothelial cells was significantly increased after VTT depletion (Fig. 3b**)**, indicating increased VEGFA signaling. In agreement with this finding, the number of filopodia-displaying endothelial cells was increased (Fig. 3c, d). Analysis of villus tip endothelial cell patterning indicated the number of endothelial cells positioned on the villus core side of the vessel was significantly decreased following VTT depletion, indicating loss of the polarized endothelial cell arrangement (Fig. 3c, e), while the total number of villus tip endothelial cells was unchanged (Fig. 3c, f). These results point to a role for VTTs in sequestering VEGFA signaling at the villus tip (Fig. 3g).

### VTTs are a distinct subepithelial fibroblast population

**Fig. 4:**
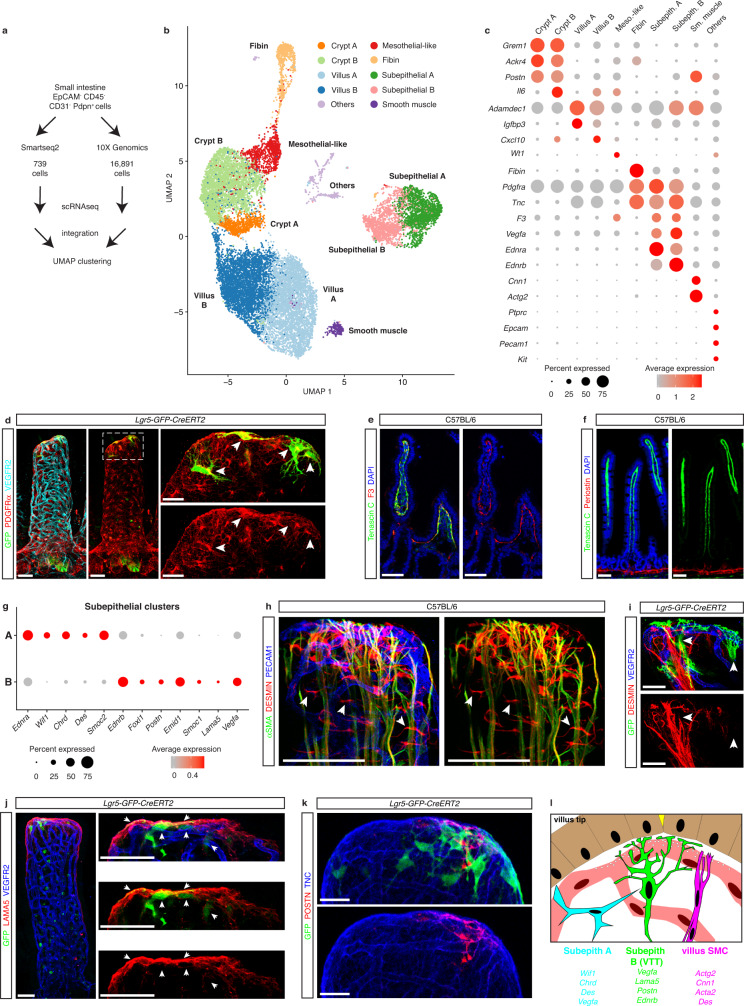
VTTs are a distinct subepithelial cell population. **a** Strategy used for single cell RNAseq (scRNAseq) of wild-type small intestinal fibroblasts by Smartseq2 and 10X Genomics technologies. **b** UMAP plot of 10 fibroblast clusters from integrated Smartseq2 and 10X Genomics scRNAseq libraries. **c** Dot plot displaying defining genes for each fibroblast cluster, color coded by expression level with dot size denoting the percent of cells in each cluster expressing the given gene. **d** VTTs are a subset of the PDGFRα^+^ subepithelial stromal network. Staining for PDGFRα (red), GFP (green) and, VEGFR2 (blood vessels, cyan). Inset: Magnification of the boxed area from (**d**), PDGFRα^+^ VTTs are highlighted by arrowheads; whole-mount immunostaining. **e** F3 (red) is expressed in all subepithelial stromal cells while TNC (green) is limited to villus fibroblasts. Paraffin section immunostaining; blue, DAPI. **f** TNC (green) and periostin (red) are expressed in the villi and crypts, respectively. Paraffin section immunostaining; blue, DAPI. **g** Dot plot of representative transcripts from subepithelial clusters A and B. **h** Desmin is produced by both subepithelial cells and villus smooth muscle cells. Staining for desmin (red) PECAM1 (blue) and αSMA (green). **i** *Lgr5*^+^ VTTs do not express desmin. Staining for GFP (green), desmin (red) and VEGFR2 (blue). (**j**) High LAMA5 expression is limited to the villus tip and VTTs. Staining for GFP (green) and LAMA5 (red). Inset, magnification of villus tip, white arrowheads denote LAMA5^+^ VTTs. Whole-mount immunostaining; blue, VEGFR2. **k** VTTs secrete POSTN at villus tip. Whole-mount immunostaining for *Lgr5*^+^ VTTs (green, GFP), POSTN (red) and TNC (blue) at the villus tip. **l** Cartoon summarizing phenotypic and typical genes expressed by villus SMCs and cells in subepithelial clusters A and B. Scale bars: 50 μm: **d**, **e**, **f**, **h**, **j**; 20 μm: **d** (inset), **i**, **j** (inset), **k**. Source data are provided as Source Data and Supplementary Data files.

To determine a mechanism promoting VTT-mediated VEGFA sequestration we sought to characterize VTTs at the molecular level and performed scRNAseq on CD45^neg^EpCAM^neg^CD31^neg^PDPN^+^ intestinal cells from wild-type adult mice (Supplementary Fig. 5a), using PDPN as a broad marker of intestinal fibroblasts^38,39^. Single cell analysis was performed using both Smartseq2 and 10X Genomics platforms and, after quality control filtering, yielded transcriptomes from 739 and 16,891 cells, respectively, which were then integrated for downstream analysis (Fig. 4a; Supplementary Fig. 5b). UMAP analysis revealed 9 main clusters consisting of mostly fibroblastic cells as well other small clusters including endothelial, epithelial and immune cells and interstitial cells of Cajal (Fig. 4b, c; Supplementary Fig. 5c; Supplementary Data file 1, https://singlecell.broadinstitute.org/single_cell/study/SCP1840). Based on cluster-defining genes, comparison with other intestinal fibroblast scRNAseq datasets and a 90-gene signature differentiating fibroblasts and mural cells^30,40–46^, we delineated villus, crypt, and subepithelial fibroblasts, smooth muscle cells and two clusters highlighted by *Fibin* and *Wt1* expression, we named *Fibin*^*+*^ and “mesothelial-like”, respectively (Fig. 4b, c; Supplementary Fig. 5c, Supplementary Data file 1).

To identify VTTs, we focused on the subepithelial fibroblast cluster, highlighted by expression of *Pdgfra*, *Tnc*, and *F3*, which contained two subsets, A and B (Fig. 4c; Supplementary Fig. 5c). PDGFRα and F3, also known as tissue factor, are markers of subepithelial cells^41,42,47,48^ and immunostaining confirmed subepithelial expression of F3 and PDGFRα in VTTs (Fig. 4d, e). Furthermore, extracellular matrix protein TNC expression is limited to the villus area (Fig. 4e, f)^7^, suggesting that the A and B subepithelial clusters were similarly restricted to villi. Differential expression analysis revealed that cluster A exclusively expressed *Wif1* and was enriched for *Chrd*, *Des*, *Smoc2* and *Ednra* while subepithelial cluster B was enriched for the intestinal telocyte marker *Foxl1*^49^ as well as *Ednrb, Postn, Smoc1*, and *Lama5* (Fig. 4g, Supplementary Data file 1). Of note, *Vegfa* expression was restricted to the subepithelial clusters (Fig. 4c), with higher expression in cluster B (Fig. 4g, Supplementary Data file 1). *Lgr5* was expressed at high levels only in “hitchhiker” epithelial cells, however analysis of raw RNA levels showed *Lgr5* co-expressed in rare *Epcam*^neg^, *Pdgfra*^+^, *Vegfa*^+^ cells (Supplementary Fig. 5d).

To confirm the identities of the two subepithelial clusters we performed whole-mount immunostaining of representative makers. scRNAseq showed *Des* expression limited to subepithelial cluster A and villus SMCs (Supplementary Fig. 5e). Immunostaining confirmed desmin co-staining with αSMA^+^ villus SMCs and revealed stellate desmin^+^ subepithelial cells aligned perpendicularly to the villus axis (Fig. 4h), consistent with prior observations^43,50^. In contrast, GFP^+^ VTTs were desmin^neg^ (Fig. 4i), indicating VTTs were not part of subepithelial cluster A.

To determine if VTTs were in subepithelial cluster B we performed immunostaining for ECM proteins LAMA5 and POSTN, which were expressed in cluster B but not cluster A (Fig. 4g, Supplementary Fig. 5f). Immunostaining showed high LAMA5 expression restricted to the villus tips, consistent with previous observations^51^ and co-expression of LAMA5 with GFP^+^ VTTs (Fig. 4j). TNC is widely expressed at the villus tip and punctate POSTN deposition was observed near GFP^+^ VTTs (Fig. 4k). As reported previously, POSTN was expressed at high levels in crypt fibroblasts (Fig. 4f)^7^. Additionally, in agreement with scRNAseq data, VEGFA was detected in both *Lgr5*-GFP-positive and -negative villus tip subepithelial cells (Supplementary Fig. 5g). In all, these results point to VTTs as a distinct population of subepithelial and perivascular fibroblasts (Fig. 4l).

### VTT-specific ADAMTS18 is necessary for constraining villus tip VEGFA signaling by limiting fibronectin accumulation

ScRNAseq analysis revealed VTTs are one of two intestinal stromal cell types expressing *Vegfa* at the villus tip, while depletion experiments showed VTTs constrain endothelial villus tip VEGFA signaling, leading to a seemingly contradictory conclusion that depleting *Vegfa*-expressing cells increases VEGFA signaling. However, VEGFA bioavailability is highly regulated by ECM binding and proteolysis^52^, and we hypothesized that VTTs sequester VEGFA through imposing a specific villus tip ECM.

Analysis of subepithelial cluster B showed that *Lama5* expression was restricted to a discrete subpopulation of cells (Supplementary Fig. 5d, 6a). Therefore, we performed UMAP clustering restricted to cells in subepithelial cluster B. Clustering revealed 3 distinct cell subsets, which we labeled 0, 1 and 2 (Supplementary Fig. 6b). Expression of *Lama5* and *Foxl1* was restricted to cluster 2, while *Vegfa* was produced by cells in all 3 clusters (Supplementary Fig. 6c). Of note, *Vegfd*, which codes for a synonymous VEGFR3 ligand, was also enriched in subepithelial cluster B2 (Supplementary Data file 2). Gene set analysis^53^ revealed cells in the subepithelial B2 cluster were highly enriched for genes coding for ECM and BM proteins (Supplementary Fig. 6e, Supplementary Data file 2), in line with our hypothesis that VTTs contribute to specific ECM and BM at the villus tip.

**Fig. 5:**
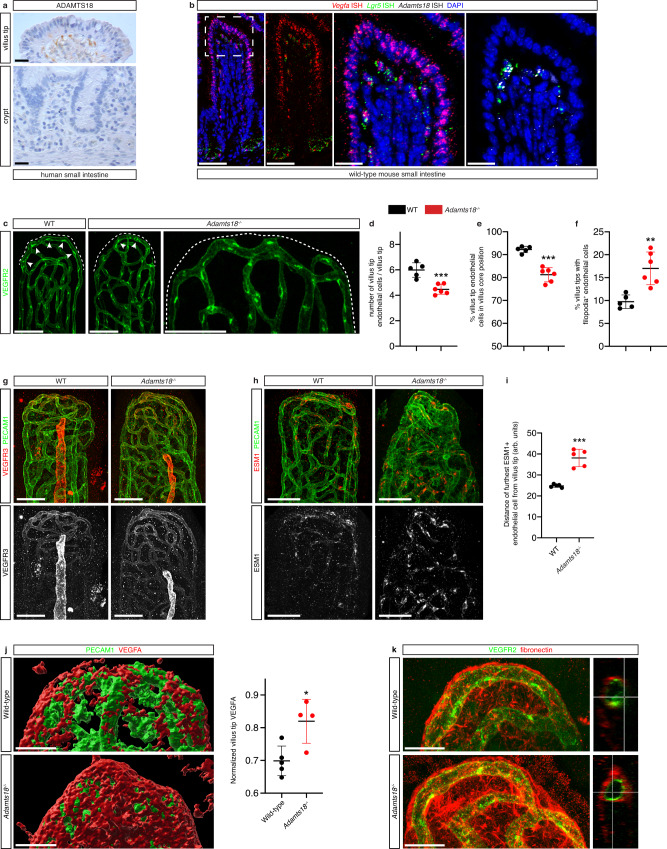
VTT-specific ADAMTS18 is necessary for constraining villus tip VEGFA signaling by limiting fibronectin accumulation. **a** ADAMTS18 expression is restricted to the villus tips in the human small intestine. Chromogenic immunostaining for ADAMTS18 (brown) of human jejunum villus tips (above) and crypts (below); H + E counterstained. **b** Co-localization of *Lgr5*, *Vegfa* and *Adamts18*. RNAscope fluorescent in situ hybridization of the intestine for *Vegfa* (red), *Lgr5* (green) and *Adamts18* (white). The white box delineates zoomed villus tip area shown in the right two panels. **c** Loss of villus tip endothelial cells and nucleus polarization in adult *Adamts18*^−/−^ mice. Whole-mount immunostaining showing decreased number of villus tip endothelial cells (white arrowheads, green perinuclear VEGFR2 immunostaining) and increased number of endothelial cell nuclei on the epithelial side of villus tip vessels (bottom). Dotted lines, epithelium. Quantification of (**d**) the number of endothelial cells/villus tip (*p* = 0.0005), (**e**) percentage of endothelial cells on the villus core side of the vessel (*p* < 0.0001), and (**f**) percentage of villus tip endothelial cells displaying filopodia in *Adamts18*^−/−^ mice and littermate wild-type controls (*p* = 0.0022); *n* = 5-6 mice. **g**–**i** Increased villus tip endothelial VEGFA signaling in *Adamts18*^−/−^ mice. Whole-mount immunostaining of villus tip vessel for PECAM1 (green) and (**g**) VEGFR3 (red, top) or (**h**) ESM1 (white bottom). **i** Quantification of distance (a.u.) at which ESM1^+^ vessels are observed from the villus tip in adult *Adamts18*^−/−^ mice or littermate wild-type controls (*p* = 0.0001, *n* = 5-6 mice). **j** Increased VEGFA at the villus tip in *Adamts18*^−/−^ mice. Wholemount immunostaining for VEGFA (red) and PECAM1 (green) at the intestinal villus tip in wild-type and *Adamts18*^−/−^ mice. Images show Imaris-generated 3D surfaces. Quantification of the VEGFA volume at the villus tip (*p* = 0.0142, *n* = 4 mice. **k** Increased deposited fibronectin at the villus tips of *Adamts18*^−/−^ mice. Wholemount immunostaining for fibronectin (red) and VEGFR2 (green) in wild-type and *Adamts18*^−/−^ mice. Scale bars: 200μm: a; 50 μm: **b**, **c**, **g**, **h**; 20μm: **b** (inset), **j**, **k**. *P < 0.05, **P < 0.01, ***P < 0.001 2-tailed unpaired Student’s *t* test. All values shown as mean ± SD. Source data are provided as a Source Data file.

Protease-driven ECM remodeling is key for promoting cell responses by allowing mechanical changes and release of signaling proteins to adapt to tissue needs^54^. Three proteases were expressed by cells in the subepithelial B2/VTT cluster, *Plau*, *Ece1*, and *Adamts18* (Supplementary Data file 2). Of the three genes, only *Adamts18* was limited to this cluster (Supplementary Fig. 6d, f) and immunostaining of human small intestine sections revealed that ADAMTS18 is restricted to subepithelial stellate-shaped villus tip cells in human small intestinal villi phenotypically resembling VTTs (Fig. 5a). We confirmed ADAMTS18 was VTT-specific by performing fluorescence in situ hybridization (FISH) for *Vegfa*, *Lgr5* and *Adamts18* and found colocalization of all three transcripts in subepithelial villus tip stromal cells (Fig. 5b). This analysis also revealed high levels of VEGFA expression in villus epithelial cells suggesting the intestine may serve as a reservoir for systemic VEGFA recently shown to promote lifespan extension^55^.

ADAMTS18 is an orphan member of the A Disintegrin And Metalloproteinase with a ThromboSpondin type 1 motif protein family^56^. It plays a key role in ECM turnover required for mammary gland stem cell maintenance^57^ and *Adamts18*^−/−^ mice display decreased carotid artery-associated ECM expression and defective basal lamina patterning^58^. We analyzed *Adamts18*^−/−^ mice to determine if ADAMTS18 plays a functional role at the villus tip. In control animals, villus tip vessels were aligned subepithelially as previously observed, however in littermate *Adamts18*^−/−^ mice villus tip vessels were detached from the subepithelial position and the number of villus tip endothelial cells was reduced (Fig. 5c, d). Additionally, endothelial cell nuclei polarization to the villus core side was disturbed in *Adamts18*^−/−^ mice (Fig. 5c, e) and the number of filopodia-displaying endothelial cells was increased (Fig. 5c, f), while length of the villus lymphatic vessels, lacteals, were unchanged (Supplementary Fig. 7a). We therefore analyzed whether *Adamts18*-deficiency altered villus tip endothelial cell VEGFA signaling. Both VEGFR3 and ESM1 were more highly expressed in *Adamts18*^−/−^ villus tip vessels than controls (Fig. 5g-i) and VEGFA protein levels were increased at the villus tip (Fig. 5j). Moreover, there was a tendency towards increased portal vein VEGFA levels in *Adamts18*^−/−^ mice compared to controls (Supplementary Fig. 7b). Taken together these data indicate that *Adamts18* deficiency leads to loss of villus tip VEGFA sequestration and an increase in VEGFA signaling, thus recapitulating the VTT ablation phenotype.

We hypothesized that loss of ADAMTS18 protease activity was driving increased bioavailability of VEGFA. ADAMTS18 cleaves fibronectin in vitro and *Adamts18*^−/−^ mice display increased fibronectin accumulation in mammary glands consistent with it being a substrate of this protease^57^. This raised our interest because fibronectin is the major ECM component binding VEGFA and significantly enhances VEGFA/VEGFR2 signaling in endothelial cells^59,60^. Interestingly, the villus tip is relatively hypoxic and acidic^6,61^ and VEGFA binds more tightly to fibronectin in low pO2/pH environments^62,63^, suggesting that fibronectin accumulation could dictate the location of VEGFA signaling. In line with this scenario, scRNAseq data showed that while *Fn1* was widely expressed in most gut fibroblasts, it was enriched in *Pdgfra*^+^ sub-epithelial fibroblasts, including those expressing *Adamts18 and Vegfa* (Supplementary Fig. 7c**)** and we confirmed that *Lgr5*-GFP^+^ VTTs are among the cells expressing fibronectin (Supplementary Fig. 7d). In wild-type mice fibronectin was mostly restricted to the epithelial side of endothelial cells, similar to VEGFA (Fig. 5j, k). However, *Adamts18*^−/−^ mice displayed increased fibronectin deposition at the villus tip, corresponding with spread of VEGFA protein (Fig. 5j, k). To confirm that ADAMTS18 degrades fibronectin we overexpressed ADAMTS18 in 293T cells (Supplementary Fig. 7e), injected them subcutaneously into immunodeficient mice and analyzed fibronectin levels by immunohistochemistry. While fibronectin was abundant in GFP-expressing tumors, it was almost absent in tumors overexpressing ADAMTS18 confirming that fibronectin is a substrate for ADAMTS18 (Supplementary Fig. 7f).

Another ADAMTS protein, ADAMTS1, directly cleaves VEGFA^64^, therefore we hypothesized that ADAMTS18 could be restricting VEGFA signaling through altering VEGFA abundance or activity. We overexpressed ADAMTS18 in myoblasts previously engineered to overexpress VEGFA (Supplementary Fig. 7g)^65^. We first assessed if myoblasts overexpressing GFP or ADAMTS18 differed in amounts of secreted VEGFA. However, the amount of VEGFA observed in the supernatants of these cell lines did not differ as measured by ELISA (Supplementary Fig. 7h). We next asked if VEGFA secreted in the presence of ADAMTS18 was functionally active. To do this we cultured the myoblasts in HUVEC basal medium and then transferred the conditioned medium to starved HUVECs and measured VEGFA signaling by western blotting for phosphorylated VEGFR2 (pVEGFR2) at different timepoints. pVEGFR2 levels were not different between HUVECs treated with the two conditioned media (Supplementary Fig. 7i) leading us to conclude that ADAMTS18 does not directly alter the capacity of VEGFA to activate angiogenic signaling in endothelial cells.

In conclusion, we present a model where ADAMTS18-producing VTTs both secrete and degrade fibronectin thereby restricting it, and VEGFA, to the epithelial side of villus tip vessels. In the absence of ADAMTS18, fibronectin spreads, increasing VEGFA signaling to the villus core side of the villus tip vessel and disturbing endothelial cell polarization.

### ADAMTS18 promotes villus tip vessel and epithelial cell integrity

**Fig. 6:**
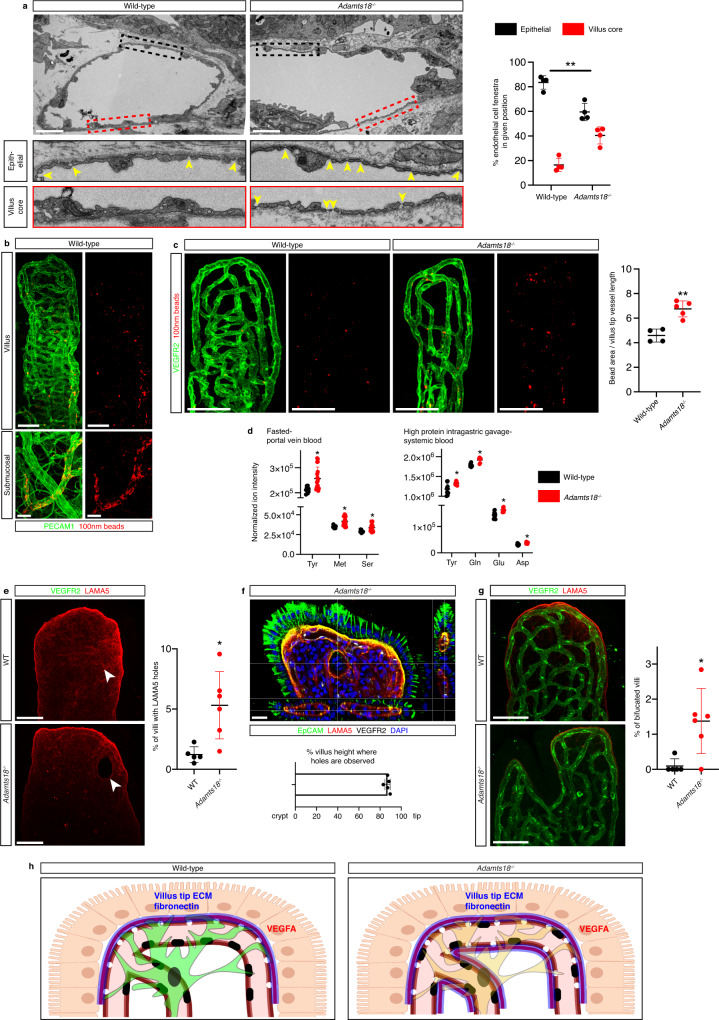
ADAMTS18 promotes villus tip vessel and epithelial cell integrity. **a** Villus tip endothelial cell fenestration extends to the villus core side in *Adamts18*^−/−^ mice. Electron micrographs of villus tip vessels from wild-type and *Adamts18*^−/−^ mice. Zoomed areas from the epithelial side (black box) or villus core side (red box) of villus tip vessels are shown below. Quantification for the percentage of fenestra observed on each side of the vessels (*p* = 0.0017, *n* = 4 mice). **b**, **c** Increased villus tip vessel leakage in *Adamts18*^−/−^ mice. **b** 100 nm fluorescent beads were injected i.p. and visualized in the intestine by wholemount imaging. In wild-type animals beads (red) accumulated sparsely throughout the villus but were concentrated around venules (green) in villi and the submucosal layer. **c** Analysis of bead distribution in the villus tip of wild-type and *Adamts18*^−/−^ mice. Quantification of the bead area/villus tip vessel area (*p* = 0.001, *n* = 4–5 mice). **d** Amino acid concentration is increased in blood of *Adamts18*^−/−^ mice. Blood was sampled from the (left) portal vein of fasted mice or (right) hearts of mice gavaged with high protein solution. (Left, Tyr, *p* = 0.0051; Met, *p* = 0.0011; Ser, *p* = 0.0063; *n* = 5–9, right, Tyr, *p* = 0.0188; Gln, *p* = 0.0005; Glu, *p* = 0.0046; Asp, 0.0018; *n* = 3–5 mice; 2 technical replicates/sample displayed). **e** Villus tip “holes” in *Adamts18*^−/−^ mice. LAMA5 staining (red) allows visualization of holes in the basement membrane. Quantification of percentage of villi with basement membrane holes in adult *Adamts18*^−/−^ mice or littermate wild-type controls (*p* = 0.0112, *n* = 5–6 mice). **f** Holes run completely through the basement membrane (red, LAMA5) at the villus tips and are covered by epithelial cells (green, EpCAM). Cross-sectional view of a villus tip hole in an *Adamts18*^−/−^ mouse; white, VEGFR2; blue, DAPI. Quantification of hole location along the villus/crypt axis, 100 denotes the villus tip. **g** Bifurcated villi are more abundant in adult *Adamts18*^−/−^ mice than littermate wild-type controls. Whole-mount immunostaining for LAMA5 (red) and VEGFR2 (green). Quantification of percentage of villi with split villi in adult *Adamts18*^−/−^ mice or littermate wild-type controls (*p* = 0.0147, *n* = 5–6 mice). **h** Scheme for proposed mechanism of VTT-derived ADAMTS18 limiting fibronectin and VEGFA to maintain the polarized endothelial cell phenotype at the intestinal villus tip (created with biorender.com). Scale bars: 50 μm: **b** (villus) **e**, **g**; 20 μm: b (submucosal), **c**, **f**; 2 μm: a. All values shown as mean ± SD. Source data are provided as Source Data and Supplementary Data files.

Local increases in VEGFA concentration lead to more endothelial fenestra^15,16^, and we hypothesized that in models of VTT loss-of-function, the number of endothelial fenestrations would be increased. Analysis of villus tip endothelial cells by transmission electron microscopy showed that while fenestrations were concentrated to the epithelial side of villus tip blood vessels in wild-type mice, fenestrations were observed on both the epithelial and villus core sides of vessels in *Adamts18*^−/−^ mice (Fig. 6a). In view of this observation, we sought to assess if the increased villus tip vessel fenestration leads to increased vessel permeability using fluorescent microspheres. We injected 100 nm fluorescent beads intravenously and visualized their distribution in the villus tip vessels 5 min later. Analysis of wild-type intestine demonstrated bead accumulation principally around venules of the villus and submucosal layer (Fig. 6b), interestingly corresponding with the site of intestinal immune cell extravasation^66^. We observed only marginal extravasation of microspheres in villus tips of control mice. However, *Adamts18*^−/−^ mice displayed prominent accumulations of fluorescent microspheres near villus tip vessels, in addition to expected extravasation near venules (Fig. 6c). In all, these data fit into a model where polarized, focused VEGFA accumulation on the epithelial side of villus tip vessels is necessary to maintain polarized fenestration. With increased VEGFA distribution in *Adamts18*^−/−^ mice, fenestration extends to the villus core side of the vessels and promotes increased vessel leakage at the villus tip.

VEGFA signaling blockade closes endothelial fenestrations and reduces glucose absorption^2,3^, providing strong evidence that fenestrations promote glucose uptake. In *Adamts18*^−/−^ mice we observed increased fenestration of villus tip endothelial cells (Fig. 6a) and we therefore hypothesized that glucose absorption could be increased in these mice. To test this we performed gavage experiments to determine the rate of glucose uptake among control and *Adamts18*^−/−^ mice. Uniformly ^13^C-labelled glucose was gavaged and the portal vein serum was sampled after 15 min. to directly analyze the absorption rate without noise from whole-body glucose metabolism (Supplementary Fig. 8a). We then analyzed the ^13^C-enrichment of metabolites in portal vein serum by untargeted metabolomics. To control for correct sampling of portal vein blood, we also sampled tail vein blood and compared levels of deoxycholic (bile) acid between the two. We found higher levels of gut-specific bile acids in portal blood and [U-^13^C]glucose only in gavage animals demonstrating a successful sampling protocol (Supplementary Fig. 8b, c; Supplementary Data files 3 and 4). Similar concentrations of fully labeled glucose were detected in *Adamts18*^−/−^ and control mice portal vein blood post-gavage (Supplementary Fig. 8d; Supplementary Data file 4) suggesting that loss of villus tip ADAMTS18 and endothelial polarization does not alter glucose absorption.

Although total protein in portal vein serum was similar between control and *Adamts18*^−/−^ mice, analysis of unlabeled metabolites indicated that *Adamts18*^−/−^ mice have a small, but significant, increase in certain amino acid levels in both portal blood of fasted mice and systemic blood of fed mice (Fig. 6d; Supplementary Fig. 8e; Supplementary Data files 5 and 6). In addition, metabolite pathway analysis showed a significant increase of amino acid metabolism intermediates and a decrease of purine metabolites (Supplementary Fig. 8f; Supplementary Data file 7). Therefore, disruption of polarized endothelial cell fenestration and villus tip stability leads to disturbed transport of some, but not all, metabolites and small molecules in the *Adamts18*^−/−^ portal vein.

In addition to alterations of endothelial cell patterning and fenestration distribution, we observed large scale disruption to villus tip epithelial cells in *Adamts18*^−/−^ mice. Earlier scanning electron microscopy studies revealed the presence of occasional villus BM holes, on average ~3 µm in diameter under homeostatic conditions^67^, and we observed these holes in control animals by LAMA5 staining (Fig. 6e). Analysis of *Adamts18*^−/−^ mice, however, revealed the presence of “donut holes” in the LAMA5^+^ BM, at least 10-fold larger, which were 5 times more frequent in *Adamts18*^−/−^ mice than littermate controls (Fig. 6e). The holes were restricted to the villus tip and distinguished by a complete lack of stroma and were covered with EpCAM^+^ epithelial cells indicating complete perforation of the villus tip (Fig. 6f). Correlating with increased holes, a significantly increased number of bifurcated villus tips were observed in *Adamts18*^−/−^ mice (Fig. 6g). In all, these results suggest VTT-specific ADAMTS18 confines VEGFA signaling and is also necessary for maintaining an intact villus tip structure (Fig. 6h).

## Discussion

Here we show that the small intestinal villus tip stromal cell unit is maintained by spatially restricted regulation of VEGFA signaling. Restriction of VEGFA to the epithelial cell-facing side of villus tip blood vessels maintains a polarized endothelial cell phenotype. VTTs are a necessary component of the stromal villus tip unit and sequester VEGFA by producing the protease ADAMTS18 to limit fibronectin accumulation, thereby limiting the spread of ECM-bound VEGFA. Therefore, we propose a model in which ADAMTS18^+^ telocytes are necessary to maintain a “just-right” level and location of VEGFA signaling in intestinal villus blood vasculature to ensure on one hand the presence of sufficient endothelial fenestrae, while avoiding excessive leakiness of the vessels and destabilization of villus tip epithelial structures.

The majority of nutrients are absorbed in the proximal small intestine and, within the villus, nutrient transport to blood circulation occurs at the villus tip^18,19^. The presence of longer villi in the proximal small intestine, together with higher villus tip hypoxia^6^ and active VEGFA signaling, suggest evolution of longer villus size, and the corresponding increase in VEGFA signaling as an adaptation for efficient nutrient absorption. VTT number is the highest in the proximal small intestine villus hence we propose that these specialized cells co-evolved with increasing villus height to promote both VEGFA expression and sequestration, allowing functional specialization of villus tip endothelial cells.

We demonstrated that sequestration of VEGFA to the epithelial side of villus tip vessels restricts fenestrations to this area. Such a villus tip vessel arrangement thus ensures that nutrients absorbed by enterocytes can be delivered quickly to the bloodstream. With increased VEGFA distribution in *Adamts18*^−/−^ mice, fenestration extends to the villus core side of the vessels and promotes increased vessel leakage at the villus tip. If endothelial fenestrations promote glucose absorption, we reasoned that the increased fenestration in *Adamts18*^−/−^ mice would increase glucose absorption. However, we did not detect a difference in glucose absorption, rather increased vessel permeability and a small increase in amino acid transport. Intestinal blood vessels have evolved to maximize nutrient uptake as most organisms contend with scare nutrients levels, therefore, given the process is already so efficient we conclude that increasing fenestrations may not influence rates of glucose uptake in a detectable manner.

In situ hybridization revealed VEGFA expression by both VTTs and intestinal epithelial cells and VEGFA was detectable in portal vein blood. Given recent findings about the systemic role of VEGFA in promoting organ health^55^, the intestine is one of the likely reservoirs for systemic VEGFA. Targeted deletion of intestinal VEGFA sources will allow analysis of whether the cellular source of VEGFA plays differing roles at the local and systemic levels.

We found that VTT-specific ADAMTS18 was necessary to maintain the villus tip vasculature through sequestering VEGFA signaling. ADAMTS proteins regulate (lymph)angiogenesis through modulation of ligand availability and remodeling of ECM^68^. ADAMTS1 limits VEGFA signaling through ligand sequestration^69^, while ADAMTS3 and ADAMTS14 are necessary for VEGFC cleavage and maturation^70–72^. We did not observe a direct role for ADAMTS18 in modulating VEGFA signaling activity in vitro. Rather our data suggest that ADAMTS18 acts to restrict fibronectin, and ECM-bound VEGF, accumulation. Hypoxia and acidity promote stronger VEGFA/fibronectin binding^62,63^. These conditions are found at the villus tip^6,61^ and likely promote a situation where the location of fibronectin accumulation dictates the area of VEGFA signaling.

Seemingly paradoxically, although villus tip endothelial cells displayed high VEGFA signaling, they were quiescent. These results are consistent with reports showing high and diffuse VEGFA signaling renders angiogenic tip cells quiescent^73–75^. Interestingly, however, these previous observations were made with sprouting postnatal retina vessels, in contrast to the non-sprouting adult villus tip endothelial cells. We speculate there are other organ-specific mechanisms, which hinder sprouting at the villus tip. *Sema3f* was specifically expressed by VTTs (Supplementary Data file 2) and its protein product is a highly anti-angiogenic factor^76^. Therefore, VTT could secrete a combination of pro- and antiangiogenic factors to maintain the polarized villus tip endothelium, while restricting cell proliferation and sprouting. One potential mechanism could be VTT directional secretion of pro- and antiangiogenic factors respectively to the epithelial and villus core side of villus tip vessels. Furthermore, the intestinal microbiota imposes a steady-state low-level inflammation in the gut^77^. The villus tip, exposed into the gut lumen, is directly exposed to this environment. Intestinal endothelial cells require TAK1 and CASP8 to prevent cell death in response to gut microbiota-derived TNFα signaling^78,79^. Therefore, VEGFA could also be playing a pro-survival role at the villus tip.

Interestingly, VEGFA signaling in endothelial cells likely affects VTT patterning. At steady-state VTTs display a highly branched, stellate shape and are positioned draping over the villus tip vessel (Fig. 2a, Supplementary Fig. 9a). However, after treatment with DC101 for 7 days we observed VTTs largely lose their stellate shape, become displaced from the perivascular location and lose their branching so the average area covered by each VTT is significantly decreased (Supplementary Fig. 9a, b). VEGFR2 is highly expressed by intestinal endothelial cells, while VTTs are VEGFR2-negative (Supplementary Fig. 9a, b), thus endothelial VEGFR2 signaling is necessary for VTT patterning. In total, these observations show a tight link exists between VTTs and VEGFA signaling at the small intestinal villus tip. Analysis of *Adamts18*-deficient *Lgr5-GFP-CreERT2* mice will also be interesting to determine if increased VEGFA signaling likewise affects VTT morphology.

Similar to other fenestrated endothelia^3,9^, villus tip endothelial cells are enriched in VEGFR3. Interestingly, VEGFR3 prevents VEGFA/VEGFR2-mediated endothelial cell-cell junction disassembly and leakage^80^, and may be playing this role at the villus tip. Furthermore, we observed that VTTs express VEGFR3 ligand *Vegfd*, suggesting that the VEGFD/VEGFR3 axis contributes to villus tip vessel junction stability.

Several ECM and BM genes enriched in VTTs (*Lama5, Smoc1 and Emid1*) are similarly expressed in developing branched organs (Supplementary Data file 2)^81–84^. These organs, such as the lung, mammary gland and kidney, develop with curved epithelial layers forming a budding tip to establish novel branches^85^. Interestingly, defects in *Adamts18*^−/−^ mice are specific to branching organs^57,86,87^. Given the molecular and cellular architecture similarities between developing branching organs and vascular defects observed in villus tips of *Adamts18*-deficient mice it is tempting to speculate that ADAMTS18-dependent budding tip blood vessel maintenance may be necessary for proper branching organ patterning. It will be also interesting to analyze whether *Adamts18*-deficiency affects angiogenesis and fenestrated vascular beds in other organs. Additionally, it will be important to characterize regional specialization of gut fibroblasts by comparing profiles of small intestinal VTTs and a recently identified population of colon crypt apex fibroblasts, which also express *Lgr5*^46^.

ADAMTS18 is expressed at the human villus tip in cells morphologically similar to VTTs. Therefore, future work should determine if the mouse villus tip stromal unit is conserved in humans. *ADAMTS18* mutations were reported in patients with Knobloch syndrome, a disease characterized by myopia and retinal degradation, however, this association is now unclear^88–91^. Therefore, future analysis of intestines from known carriers of *ADAMTS18* mutations could be informative.

A recent study reported that *Lgr5*^+^ VTTs orchestrate villus tip epithelial gene expression^30^. Here, detailed scRNAseq analysis allowed identification of specific villus tip stromal cells, revealed a novel mechanism of villus tip maintenance and uncovered an unexpected function of VTTs in the regulation of intestinal vasculature. Analysis of the 3D cellular interactome demonstrated that VTTs simultaneously communicate with epithelial, immune, and other stromal cells. Given our current results, we propose that VTTs could provide a signaling link between epithelial cells and the stroma, including villus tip endothelial cells. VTTs could therefore form one chain of a villus tip epithelial-VTT-endothelial unit that promotes nutrient uptake. Future work will determine if other intestinal processes are affected by this villus tip unit.

## Methods

### Animal models

Animal experiments were approved by the Animal Ethics Committee of Vaud, Switzerland. *Lgr5-EGFP-CreERT2*^29^, *mTmG*^92^, *Rosa26-DTA*^93^, *Esm1-CreERT2*^94^ and *Adamts18*^−/−^ ^87^ mice were previously described. C57BL/6 J mice were purchased from Janvier Labs. All mice were maintained on a C57Bl/6 background. Experiments were performed with age- and sex-matched cohorts of 8–12-week-old mice (unless otherwise noted) and consisted of males and females in approximately equal numbers. Mice were provided water and food (Scientific Animal Food & Engineering, R150) ad libitum. Mice were on a 12 hour light/dark cycle and kept at 22 °C ± 2 °C with relative humidity of 55% ± 10%. Cre recombinase was activated in 8-12-week-old mice by intraperitoneal (i.p.) injection of 50 mg/kg of tamoxifen in sunflower oil (Sigma). VEGFR2 blocking antibody DC101 was described previously^25^. DC101 and control rat anti-horseradish peroxidase IgG (BioXCell) were delivered by i.p. injections (40 mg/kg) every 2 days for 7 days. Pimonidazole (60 mg/kg; Hypoxyprobe) was injected i.p. 30 min. prior to sacrifice. For fluorescent bead analysis, 100μl of 100 nm beads (580/605 FluoSpheres, Molecular Probes) diluted 1:5 in PBS were injected i.v. and mice were euthanized after 5 min.

### Mouse tissue collection, staining procedures and image acquisition

Whole-mount and paraffin section immunostaining was performed as previously described^32^. Intestinal villus images from Figs. 1f and 4h had epithelial cells removed prior to imaging. Primary antibodies are listed in Supplementary Table 1 and were resuspended according to manufacturers’ recommendations when supplied lyophilized. Alexa Fluor 488, 555, and 647 fluorochrome-conjugated secondary antibodies (Invitrogen) were used for signal detection. Nuclei were detected with DAPI. For FISH, probes were purchased from RNAscope (Mm-Lgr5-C3; Mm-Vegfa-ver2-C3; Mm-Adamts18) and hybridization was performed according to manufacturer’s recommendations. Confocal images were obtained using Zeiss LSM 780, Zeiss LSM 510 META or Leica SP5 TANDEM microscopes and standard fluorescent images were obtained using a Zeiss Axio Imager Z1. Images were analyzed using Imaris (Bitplane), ImageJ (NIH) and Photoshop (Adobe) software.

### Human tissue collection, staining procedures and image acquisition

Use of anonymous human intestine samples was approved by the Canton of Vaud Ethical Committee under project 2016-01834. Patients signed consent for use of samples for research. Chromogenic antigen detection on formalin-fixed paraffin sections was performed with antibodies listed in Supplementary Table 1 as previously described^95^. Bright field images were acquired using a Zeiss Axio Imager Z1.

### Intestinal fibroblast sorting

Intestinal fibroblast sorting was performed as previously described^96^. Mice were sacrificed and the intestine was dissected and flushed with ice-cold PBS. Peyer’s patches were removed and the intestine was cut into 1 cm pieces, which were put in a 10 mM EDTA DMEM solution agitating at 37 °C for 30 min. to remove epithelial cells. The remaining tissue was digested thrice with Liberase TL (192.5 µg/mL, Roche) in DMEM (Gibco) containing 2% FBS, CaCl_2_ (2 mM) and 50ug/ml DNase I with constant stirring at 37 °C for 20 min and washed with medium. The cell suspension was incubated with labeled antibodies listed on Supplementary Table 1.

### SmartSeq2 scRNAseq

Smartseq2 scRNAseq was performed as previously described^97^. Briefly, live, single EpCAM^−^CD45^−^CD31^−^PDPN^+^ cells were sorted using a FACSAria III (BD Biosciences) into 2.3 μl lysis buffer pre-dispensed in a 384-well plate using the single cell mask. All FACS data were analyzed using FlowJo v10.1 (FlowJo) and FACSDiva v8.0.2 (BD Biosciences).

Single-cell libraries were prepared as described previously^98^. 0.0025 μl of a 1:40,000 diluted ERCC spike-in concentration stock was used, and all cDNA was amplified with 22 PCR cycles before QC control with a Bioanalyzer (Agilent Biosystems). The libraries were sequenced on a HiSeq2500 (Illumina) with single 50-bp reads (dual indexing reads). After demultiplexing with bcl2fastq (bcl2fastq −p 56—output-dir lane_$lane), SMART-seq2 based FASTQ files were trimmed with Trim Galore (v0.4.4) followed by alignment of the data to the mouse reference genome (mm10-GRCm38) using TopHat (v2.1.1) and bowtie2 (v2.2.6.0). PCR duplicates were removed using SAMtools (v0.1.18). Counting of fragments aligning per gene was done using the featurecounts function of the Subread package (v1.4.6-p5).

### 10X Genomics scRNAseq

Live, single EpCAM^−^CD45^−^CD31^−^PDPN^+^ cells were sorted using a MoFlo Astrios EQ (Beckman Coulter) and processed for single cell libraries according to 10X Genomics recommendations. Libraries were sequenced on a HiSeq4000 (Illumina) and count tables were exported with Cell Ranger software (10X Genomics). Sequencing data were aligned to the mouse reference genome (mm10-GRCm38) and processed using the Cellranger 3.1.0 pipeline (10X Genomics) with parameters—chemistry SC3Pv3.

The raw gene expression matrix from both sequencing techniques were filtered and normalized using the Seurat R package^99^. The following criteria were used: cells with >10 genes expressed and genes with >200 cells expressing it and <20% of mitochondrial gene expression in UMI counts were kept. From the filtered cells, the gene expression matrices were normalized to the total UMI counts per cell and transformed to the natural log scale. Using the integration in Seurat R we merged the two datasets using as anchors the 4000 most variable genes using the FindVariableGenes function. Principal component analysis was performed using the variably expressed genes; the number of principal components was selected from the knee point of the elbow plot. Resolution parameters in function FindClusters from 0.2 to 0.6 were explored for better subcluster representation.

### Gene set analysis

The subepithelial cluster B2 gene list from integrated fibroblast scRNAseq data was used to query the MSigDB (v.7.1) using the “Investigate Gene Sets” function at the Gene Set Enrichment Analysis website (gsea-msigdb.org; UC San Diego and Broad Institute)^53^. Overlaps were computed using the “Canonical pathways” and GO “cellular component” gene sets. Only results with an FDR q-value <10^−10^ are displayed.

### Blood sampling

Mice were fasted for 6 hours prior to all blood sampling. For glucose absorption analysis 10μl/g mouse of U^13^C-glucose (0.5 M, Sigma) or vehicle (saline) was gavaged and allowed to be absorbed for 15 min. Mice were then anesthetized and the portal vein blood was sampled by cutting the portal vein with small scissors. For re-feeding experiments mice a commercially available whey-based protein supplement (QNT) suspended in saline was gavaged at 1.56 g/kg mouse and allowed to be absorbed for 30 min. Mice were then anesthetized and systemic blood was sampled from the heart. For both experiments serum was frozen until analysis.

### Metabolomic analysis

Polar metabolites were extracted from 20 μl of plasma with 180 μl of room temperature 80% methanol. Untargeted metabolomics of extracts was done by flow injection analysis–time-of-flight mass spectrometry on an Agilent 6550 Q-TOF instrument as previously described^100^. Mass spectra were recorded from a mass/charge ratio of 50 to 1000 in high-resolution negative ionization mode. Ions were annotated by matching their measured mass with reference compounds derived from the Human Metabolome Database (HMDB 4.0), allowing a tolerance of 1 mDa. Significance was calculated using Student’s *t* test and corrected for multiple hypothesis testing with the BH method^101^. Metabolites with a *p* value < 0.05 and abs(log2(FC)) > 0.25 were selected to perform enrichment analysis, followed by multiple hypothesis testing correction with the BH method^101^. All statistical analyses were performed in MATLAB R2020b (The MathWorks) with an in-house developed pipeline. Ion intensities data are provided in Supplementary Data files 3, 4 and 6.

### Cell culture, lentiviral overexpression and tumor experiments

293 T cells (ATCC) were cultured in DMEM supplemented with 10% FBS and antibiotics. Lentiviral vector cloning and lentivirus preparation for GFP and human ADAMTS18 were performed by VectorBuilder. 293 T cells were infected with lentivirus at an MOI of 2 and infected cells were purified by puromycin selection. Cells were grown to 70% confluence and 10^6^ cells were injected subcutaneously into *Nod/Scid/Il2rγ*^−/−^ mice (in house breeding facility). Mouse myoblast cells expressing murine VEGFA and dsRed^65,102^ were previously described and cultured on collagen-coated dishes (Sigma) in DMEM supplemented with 20% FBS, 2.5 ng/ml bFGF (Peprotech) and antibiotics. Cells were infected with GFP and human ADAMTS18 lentivirus at an MOI of 2 and infected cells were purified by puromycin selection.

### ELISA

Mouse serum VEGFA protein levels were measured using the VEGFA ELISA Kit, Mouse (Merck Millipore) according to manufacturer’s recommendations.

### Tissue preparation for block-face scanning electron microscopy

Ten-week-old Lgr5-EGFP-CreERT2 mice were perfused, via the heart, with a buffered mix of 2.5 % glutaraldehyde and 2.0 % paraformaldehyde in 0.1 M phosphate buffer (pH 7.4). The small intestine was removed and embedded in 5% agarose, and 80 µm thick, transverse sections, cut with a vibratome. After confocal microscopy was used to capture images of the GFP labelled villi the sections were stained and embedded for block-face scanning electron microscopy. The sections were post-fixed in potassium ferrocyanide (1.5%) and osmium (2%), then stained with thiocarbohydrazide (1%) followed by osmium tetroxide (2%). They were then stained overnight in uranyl acetate (1%), washed in distilled water at 50 °C, before being stained with lead aspartate at the same temperature. They were finally dehydrated in increasing concentrations of ethanol and then embedded in Spurr’s resin and hardened at 65 °C for 24 h between glass slides.

### Tissue preparation for transmission electron microscopy

For transmission electron microscopy, mice were perfused via the heart with the same fixative as above (2.5 % glutaraldehyde and 2.0 % paraformaldehyde in 0.1 M phosphate buffer, pH 7.4), and sliced in the same way. They were then postfixed 1.0 % osmium tetroxide with 1.5% potassium ferrocyanide, and then 1.0% osmium tetroxide alone. They were finally stained for 30 minutes in 1% uranyl acetate in water before being dehydrated through increasing concentrations of alcohol and then embedded in Durcupan ACM (Fluka, Switzerland) resin before being placed between glass slides and the resin cured. Once hardened, the regions of interest were cut away from the rest of the tissue sections, glued to blank resin blocks with cyanocrylate glue, and thin (50 nm thick) sections cut with a diamond knife. These were collected onto pioloform support films on single-slot copper grids, contrasted with lead citrate and uranyl acetate.

### Block face scanning electron microscopy

To collect serial electron microscopy images of the region containing the GFP labelled cells, images of the section before and after resin embedding were superimposed. This indicated the regions where fluorescence was present, and this part was trimmed from the rest of the section using a razor blade and glued to an aluminium stub using conductive glue. Further trimming with a glass knife produced a small (approximately 300 × 300 µm) block that was then mounted inside a scanning electron microscope (Zeiss Merlin, Zeiss NTS) holding a block face cutting microtome (3View, Gatan). Layers of resin, 50 nm thick, were cut from the block surface, and sequential images collected after each layer was removed. An acceleration voltage of the 1.7 kV was used with a pixel size of 7 nm with a dwell time of 1 µs. Series nearly aligned images were collected and aligned in the FIJI imaging software (www.fiji.sc). Segmentation of different cells and structures were carried out in the FIJI software running the TrakEM2 plugin. Models were exported to 3D modelling software (Blender.org) for analysis.

### Transmission electron microscopy

Areas of interest were imaged with a transmission electron microscope JEOL JEM-2100Plus (JEOL) at an acceleration voltage of 80 kV with a TVIPS TemCamXF416 digital camera (TVIPS) using EM-MENU software (TVIPS).

### Statistics and Reproducibility

Two-tailed unpaired Student’s *t* tests were performed to determine statistical significance between two means. Data are shown as mean ± SD. Results shown as representitive images were from at least two independent experiments.

## Supplementary information

Supplementary Information File

Description of Additional Supplementary Files

Supplementary Dataset 1

Supplementary Dataset 2

Supplementary Dataset 3

Supplementary Dataset 4

Supplementary Dataset 5

Supplementary Dataset 6

Supplementary Dataset 7

Supplementary Movie 1

## Data Availability

The data supporting the findings from this study are available within the manuscript and its supplementary information. Intestinal fibroblast scRNAseq data were deposited in the Gene Expression Omnibus (GSE154821) and Broad Institute Single Cell Portal (SCP1840). Metabolomic data were deposited in massIVE (accession number MSV000089080). Source data are provided with this paper.

## Source data

Source Data
