# Supplementary material for: ADAMTS18+ villus tip telocytes maintain a polarized VEGFA signaling domain and fenestrations in nutrient-absorbing intestinal blood vessels
Source: Nat Commun. 2022 Jul 9;13:3983. doi: 10.1038/s41467-022-31571-2 (PMC9271081; doi:10.1038/s41467-022-31571-2)
Supplement: Supplementary file 1 — Supplementary Information File [file 41467_2022_31571_MOESM1_ESM.pdf]

Supplementary Figure 1

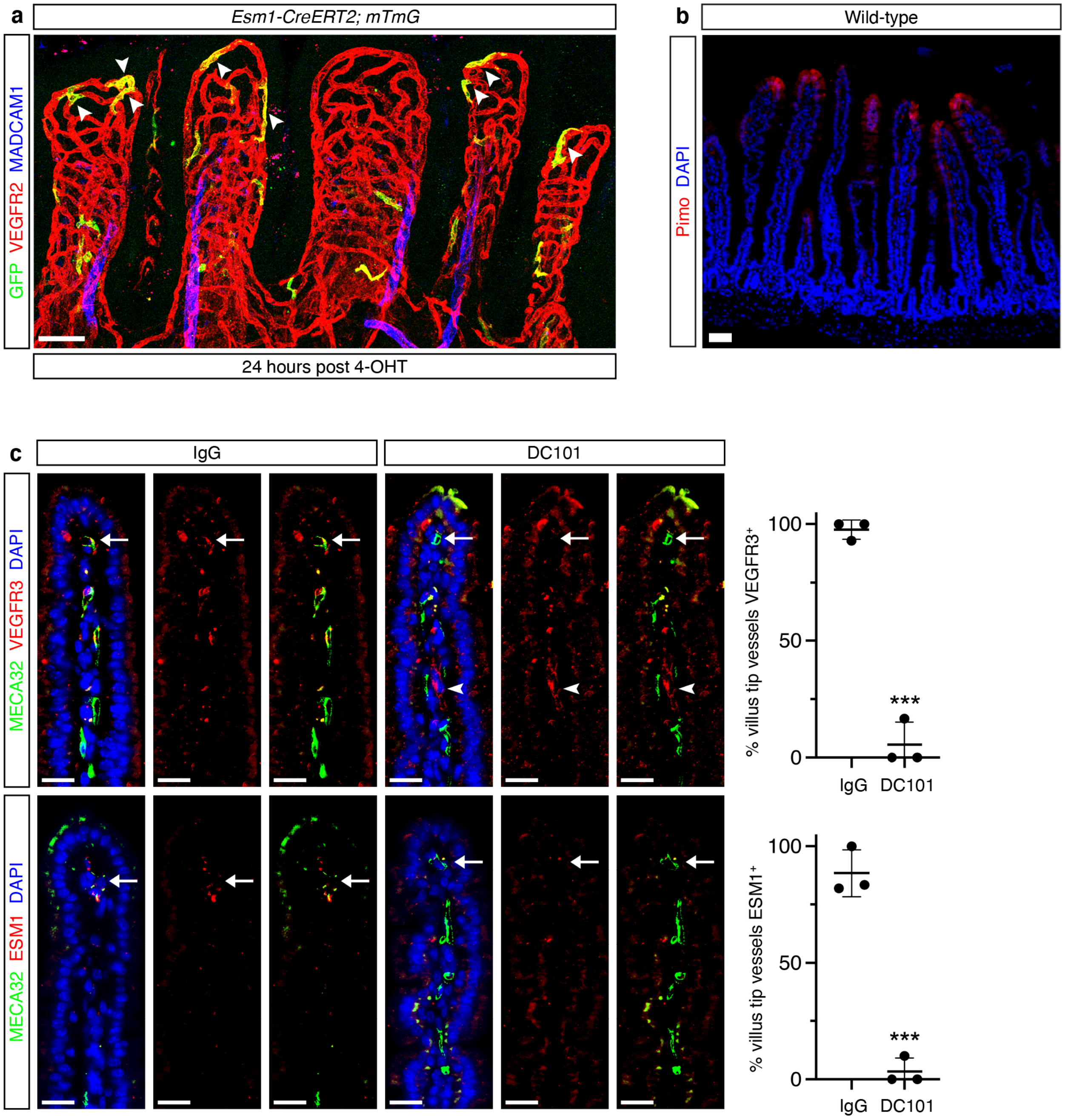

Supplementary Figure 1: High VEGFA signaling at the small intestinal villus tip. (a) Wholemount immunostaining for GFP (green), VEGFR2 (red) and MADCAM1 (blue) in the intestine of *Esm1-CreERT2; mTmG* mice 24 hours after 4-OHT injection. (**b**) Small intestinal villus tips are hypoxic. Paraffin section immunostaining for pimonidazole (Pimo, red) in the jejunum of an adult C57BL/6J mouse. (**c**) VEGFA signaling blockade decreases villus tip vessel

expression of ESM1 and VEGFR3. Paraffin section staining for VEGFR3 (red, top) or ESM1 (red, bottom) and vessels (green, MECA32) in intestinal villi from mice treated with control or VEGFR2 blocking antibodies (DC101). Quantification of number of villus tip VEGFR3+ (top, p=0.0001) or ESM1+ (bottom, p=0.0002) endothelial cells (mean ± SD); *n* = 3. Arrows: villus tip vessels; arrowheads VEGFR3^{+} lymphatic vessel. Scale bars: 50 µm:a, b; 20 µm: c. Source data are provided as a Source Data file.

Supplementary Figure 2

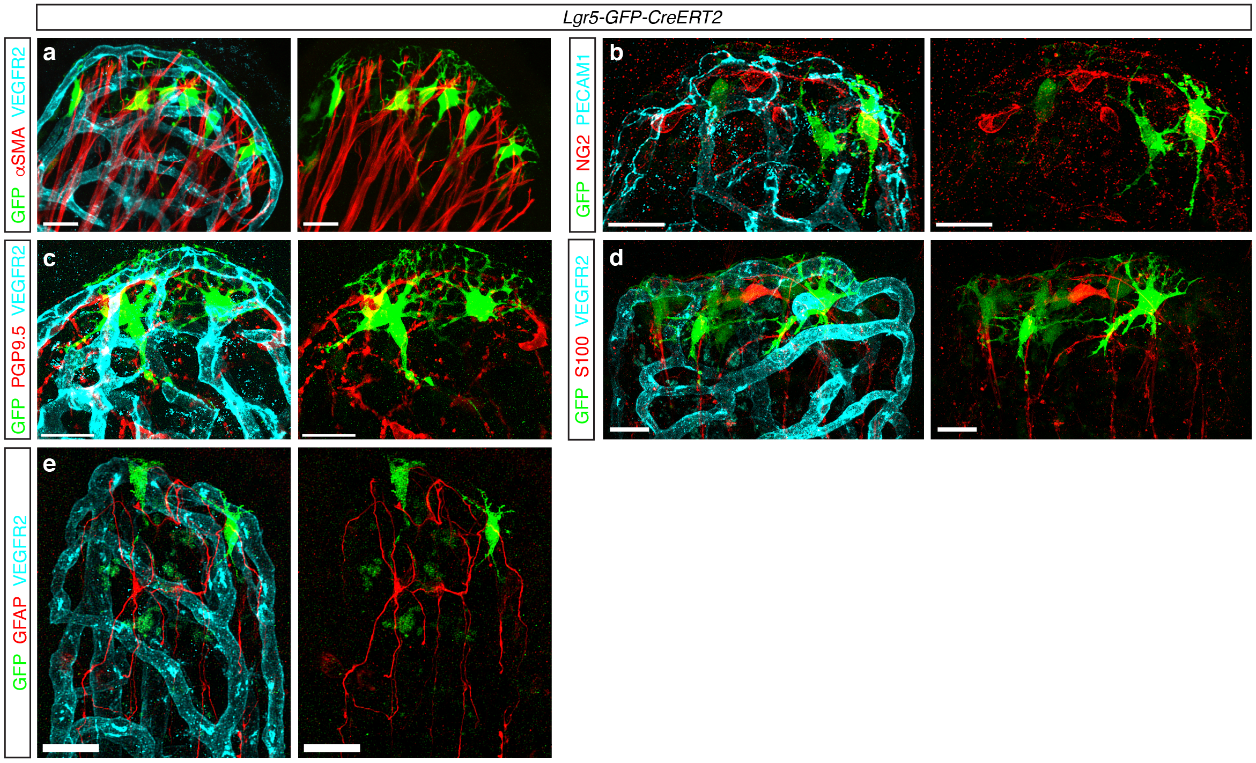

Supplementary Figure 2: VTTs are distinct from other villus tip perivascular cells. (**a**) VTT cell bodies (GFP, green) are aligned with villus smooth muscle cells (SMCs; red, αSMA) however are αSMA^{neg}; blood vessels (cyan, VEGFR2). (**b**) VTTs (green, GFP) do not express NG2 (red); blood vessels (cyan, PECAM1). (**c-e**) VTTs are negative for the neuronal and glial markers PGP9.5, S100 and GFAP. VTTs (green, GFP), blood vessels (cyan, VEGFR2). (**c**) PGP9.5 (red). (**d**) S100 (red). (**e**) GFAP (red). Scale bars: 20 µm.

Supplementary Figure 3

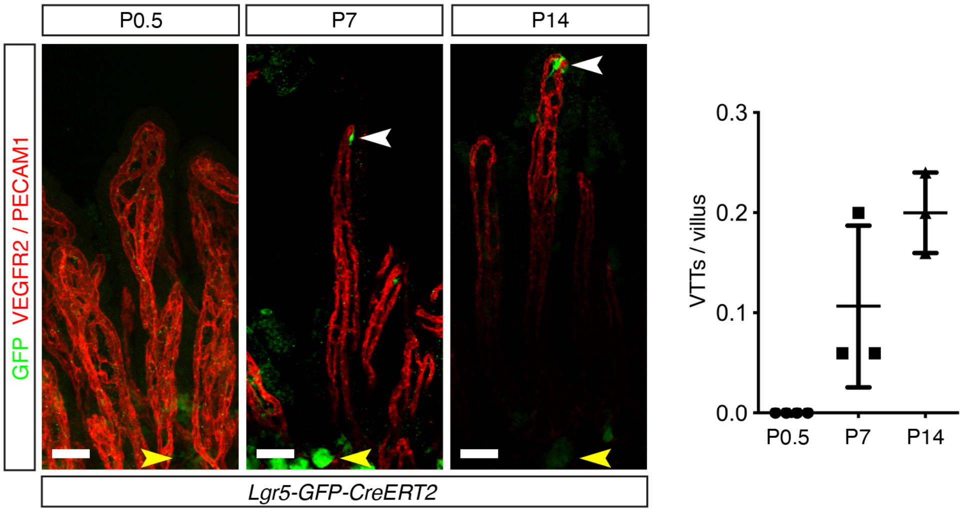

Supplementary Figure 3: VTTs are observed at the villus tip postnatally. (**a**) VTTs (green, GFP, white arrowheads) are first observed postnatally at villus tips and their numbers increase with age. Intestinal stem cells, yellow arrowheads; blood vessels (red, VEGFR2). Quantification of number of VTTs per villus at P0.5, P7 and P14 (mean ± SD); n = 3-4. Scale bars: 20 µm. Source data are provided as a Source Data file.

Supplementary Figure 4

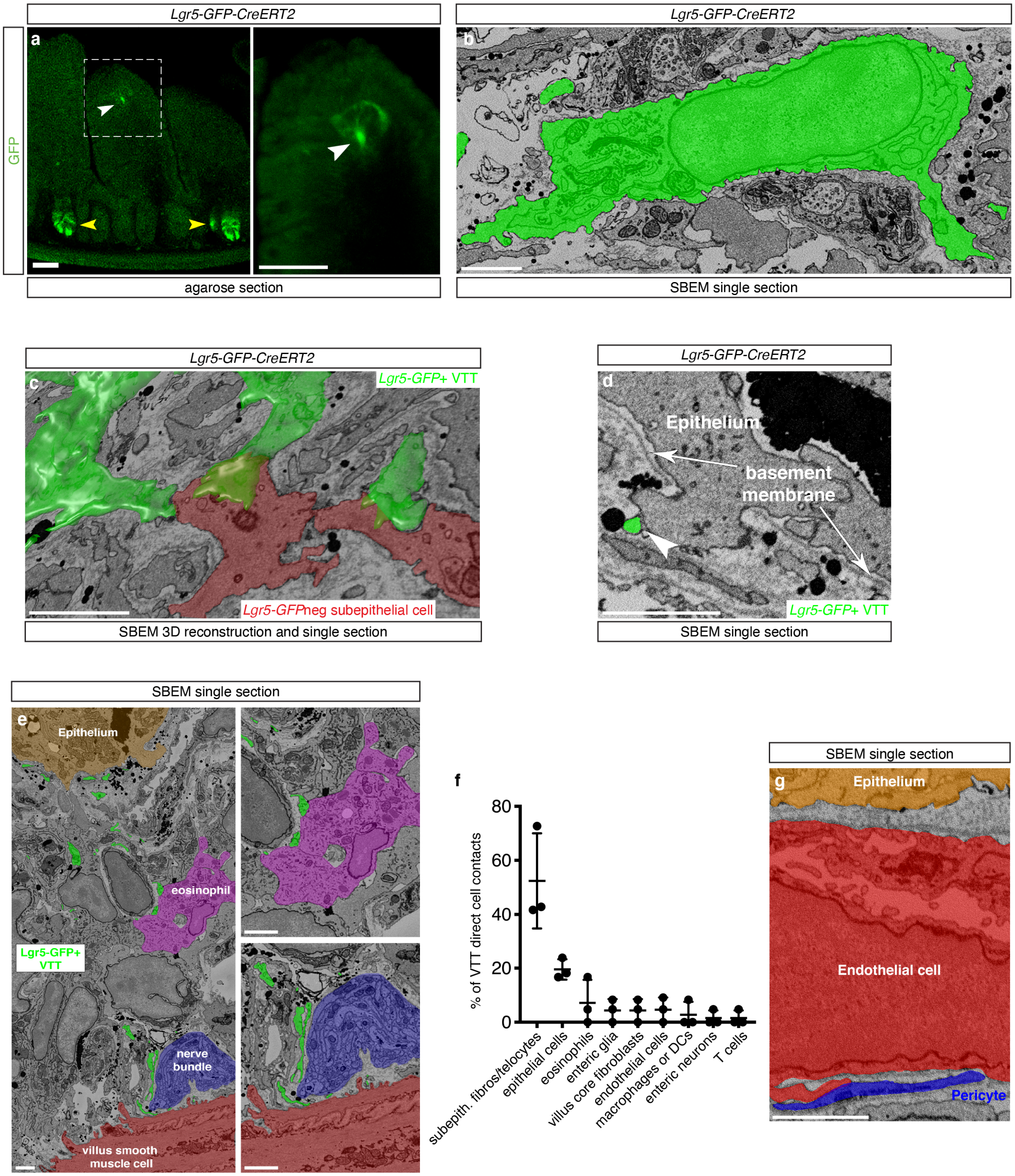

Supplementary Figure 4: Ultrastructural analysis of VTT cell-cell interactions and microenvironment. (**a**) VTTs (green, white arrowhead) are distinguished by cell morphology and villus tip location from intestinal epithelial stem cells (yellow arrowheads) in agarose sections.

Inset: increased magnification of boxed area. (**b**) VTT cell body (green) containing mitochondria and endoplasmic reticula. The nucleus contains a mix of hetero- and euchromatin (**c, d**) The majority of VTTs (green) directly contact (**c**) other subepithelial cells (red) or (**d**) intestinal epithelial cells. (**e**) The minority of VTT (green) contacts are with cells in the villus core, such as eosinophils (magenta), villus smooth muscle cells (red) and nerves (blue). (**f**) Quantification of the percentage of direct VTT contacts with given cell types (mean ± SD); $n = 3$. (**g**) Endothelial cells (red) make direct contacts with pericytes (blue); epithelium (orange). Scale bars: 50 µm: a; 2 µm: b-e, g. Source data are provided as a Source Data file.

Supplementary Figure 5

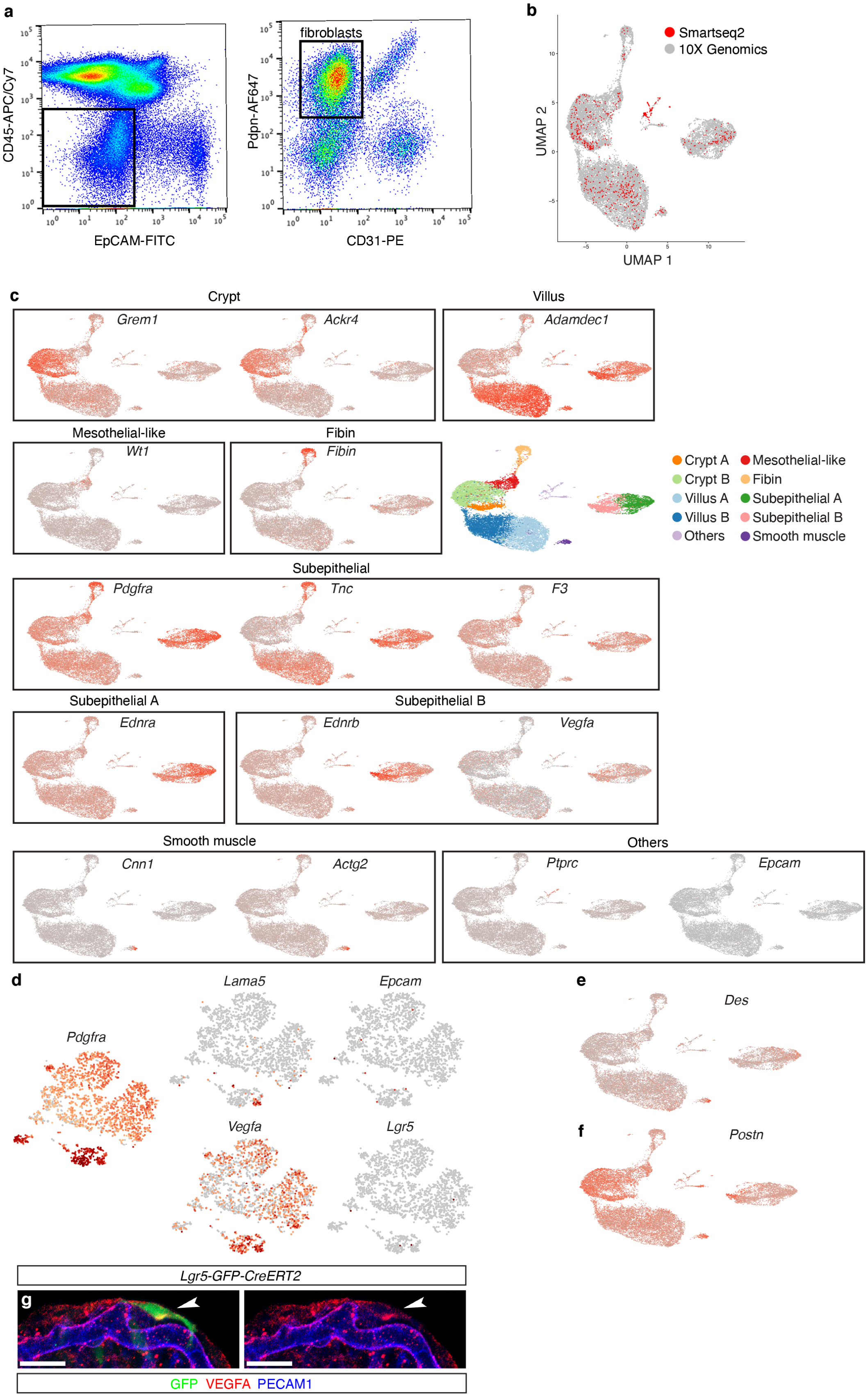

Supplementary Figure 5: Analysis of small intestinal fibroblast single cell RNAseq data. (**a**) Gating strategy for isolation of intestinal fibroblasts by flow-cytometry-based cell sorting. Single, live CD45^{neg}, EpCAM^{neg}, CD31^{neg}, PDPN^{+} cells were sorted. (**b**) UMAP projection of integrated Smartseq2 and 10X Genomics scRNAseq clustering with Smartseq2-sorted intestinal fibroblasts indicated in red. (**c**) UMAP projections with specific gene expression for the main defined fibroblast clusters. (**d**) tSNE plots of 10X Genomics scRNAseq data of *Lgr5* expression in *Pdgfra*$^{+}$, *Vegfa*$^{+}$, *Lama5*$^{+}$, *Epcam*^{neg} cells. (**e**) *Des* (desmin) expression is limited to subepithelial cluster A and SMCs. (**f**) *Postn* (periostin) expression is enriched in crypt fibroblast cluster but also expressed in the subepithelial cluster B. (**g**) VTTs (green, GFP) express VEGFA (red) at the villus tip. Scale bars: 50 µm. Source data are provided as Source Data and Supplementary Data files.

Supplementary Figure 6

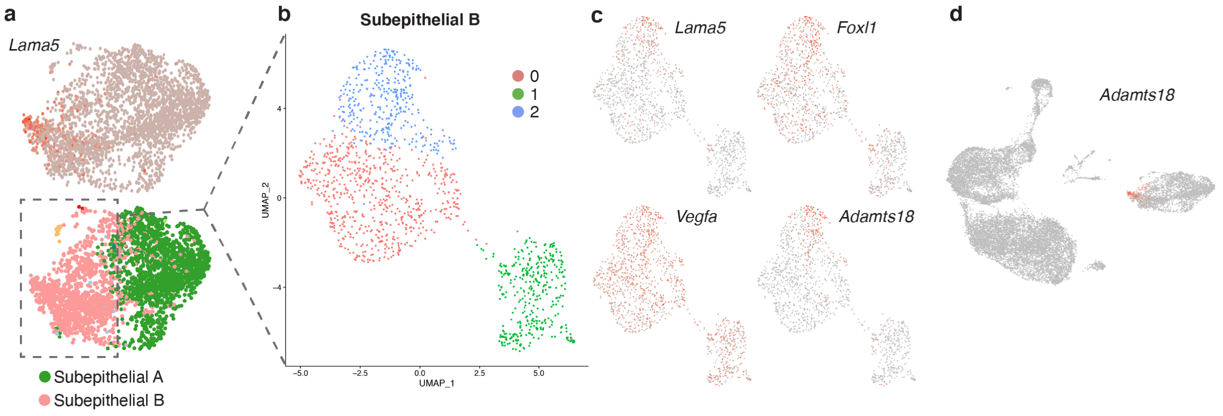

e

CLUSTER Subepithelial B2

GSEA_CANONICAL_PATHWAYS

Gene Set Name	# of genes	FDR q-value	genes
NABA_MATRISOME	37	7.7 x 10^{-26}	*Adamts18, Agt, Bmp2, Bmp7, Col12a1, Col13a1, Col3a1, Col4a5, Cspg4, Emid1, Emilin1, Fgf9, Fst, Htra1, Igsf10, Inhba, Lama4, Lama5, Lamb1, Npnt, Nrg1, Ntf3, Papln, Pcsk6, Plau, Postn, Sema3f, Sema6a, Smoc1, Sparcl1, Srpx2, Tgfbi, Tnc, Vegfa, Vegfd, Vwa1, Wnt5a*
NABA_CORE_ MATRISOME	19	2.7 x 10^{-17}	*Col12a1, Col13a1, Col3a1, Col4a5, Emid1, Emilin1, Igsf10, Lama4, Lama5, Lamb1, Npnt, Papln, Postn, Smoc1, Sparcl1, Srpx2, Tgfbi, Tnc, Vwa1*
NABA_ECM_ GLYCOPROTEINS	15	4.7 x 10^{-14}	*Emid1, Emilin1, Igsf10, Lama5, Lamb1, Npnt, Papln, Postn, Smoc1, Sparcl1, Srpx2, Tgfbi, Tnc, Vwa1*
PID_INTEGRIN1_ PATHWAY	11	8.2x 10^{-14}	*Col3a1, Col4a5, Cspg4, Lama4, Lama5, Lamb1, Npnt, Plau, Tgfbi, Tnc, Vegfa*
KEGG_FOCAL_ ADHESION	12	7.0 x 10^{-10}	*Cav1, Ccnd1, Col3a1, Lama4, Lama5, Lamb1, Myl9, Mylk, Pdgfra, Tnc, Vegfa, Vegfd,*

GSEA_GO_CELLULAR_COMPARTMENT

Gene Set Name	# of genes	FDR q-value	genes
GO_EXTRACELLULAR _MATRIX	29	7.5 x 10^{-25}	*Agt, Bmp7, Cdh13, Col12a1, Col13a1, Col3a1, Col4a5, Cspg4, Emid1, Emilin1, F3, Fgf9, Htra1, Lama4, Lama5, Lamb1, Nav2, Npnt, Pcsk6, Postn, Smoc1, Sparcl1, Srpx2, Tgfbi, Tnc, Tril, Vegfa, Vwa1, Wnt5a*
GO_COLLAGEN_CONTAINING_ EXTRACELLULAR_MATRIX	26	5.3 x 10^{-24}	*Agt, Bmp7, Cdh13, Col12a1, Col13a1, Col3a1, Col4a5, Cspg4, Emilin1, F3, Fgf9, Htra1, Lama4, Lama5, Lamb1, Nav2, Npnt, Pcsk6, Postn, Smoc1, Sparcl1, Srpx2, Tgfbi, Tnc, Vwa1, Wnt5a*
GO_BASEMENT_MEMBRANE	10	1.5 x 10^{-10}	*Col4a5, Fgf9, Lama4, Lama5, Lamb1, Npnt, Smoc1, Tgfbi, Tnc, Vwa1*

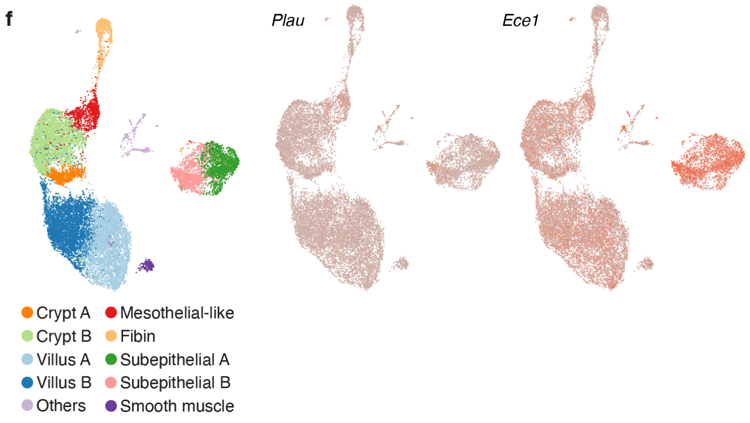

Supplementary Figure 6: Analysis of VTT-containing scRNAseq cluster "subepithelial B2". (**a**) *Lama5* is restricted to a subset of cells from subepithelial cluster B. UMAP projections of A and B subepithelial clusters. (**b**) Analysis of subepithelial cluster B reveals three distinct clusters denoted as 0, 1 and 2. (**c**) *Lama5* and *Adamts18* are limited to subepithelial cluster B2, while *Vegfa* and *Foxl1* are homogenously expressed among the three clusters. (**d**) *Adamts18* is uniquely expressed in subepithelial cluster B2. UMAP projection of all clusters with *Adamts18* expression highlighted. (**e**) Gene set analysis of transcripts expressed in subepithelial cluster B identified by scRNAseq. (**f**) *Plau* and *Ece1* are broadly expressed in small intestinal fibroblasts. UMAP plots showing cells expressing the given genes. Source data are provided as Source Data and Supplementary Data files.

Supplementary Figure 7

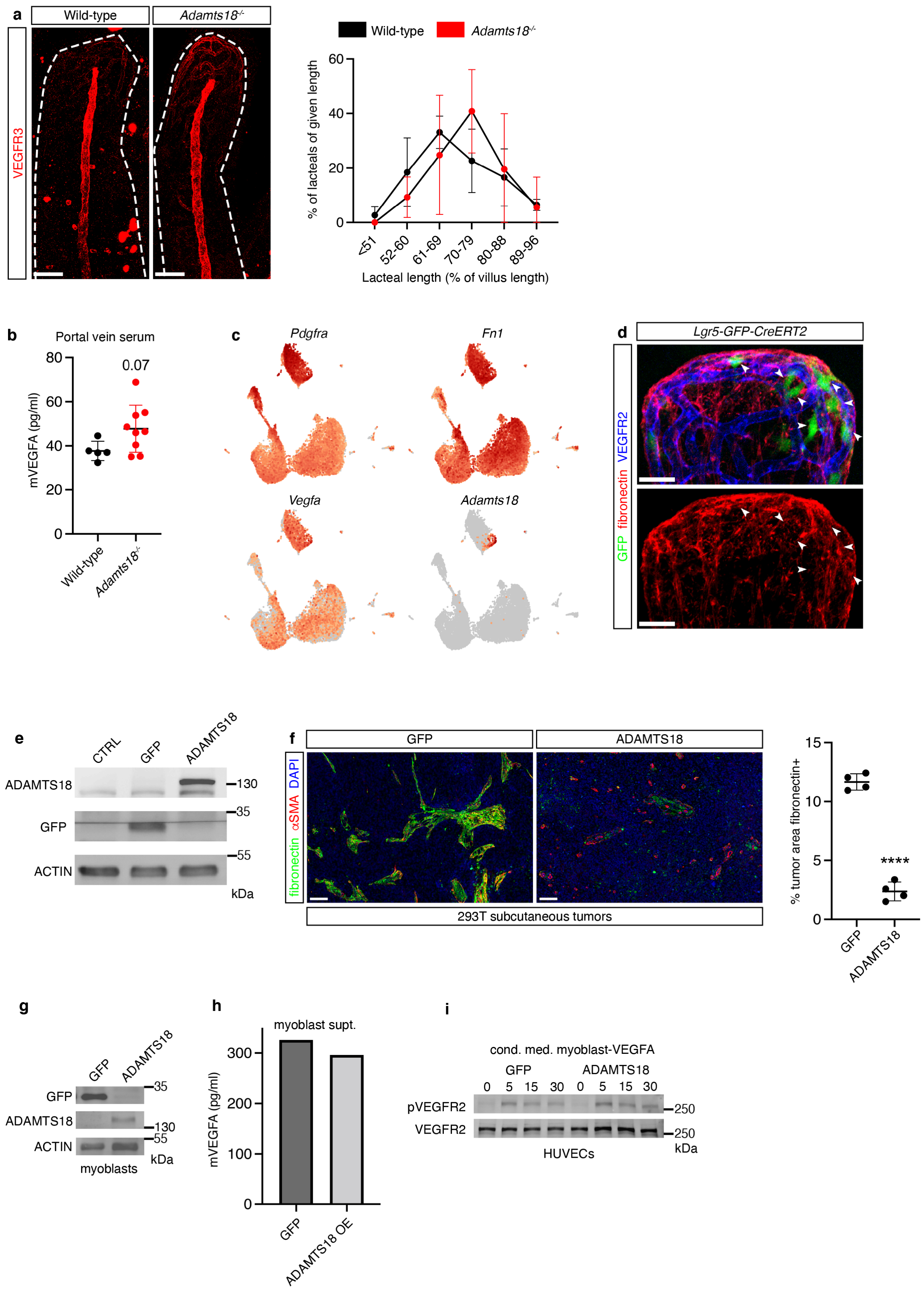

Supplementary Figure 7: Functional analysis of ADAMTS18 on lacteal patterning, extracellular matrix digestion and endothelial cell VEGFA signaling. (**a**) No difference in lacteal length in *Adamts18*$^{-/-}$ mice. Wholemount immunostaining for VEGFR3 and quantification of the binned lacteal length in wild-type and *Adamts18*$^{-/-}$ mice. (**b**) Quantification of portal vein serum VEGFA by ELISA from wild-type and *Adamts18*$^{-/-}$ mice, n=5-9. (**c**) *Adamts18*$^{+}$ VTTs express *Fn1*. UMAP plots of intestinal fibroblast scRNAseq data showing expression of *Pdgfra*, *Fn1*, *Vegfa* and *Adamts18*. (**d**) VTTs express fibronectin. Wholemount immunostaining at the villus tip of *Lgr5-GFP-CreERT2* mice for GFP (green), fibronectin (red) and VEGFR2 (blue). (**e**) Lentivral overexpression of GFP and ADAMTS18 in 293T cells. (**f**) ADAMTS18 overexpression decreases tumor fibronectin deposition. GFP- and ADAMTS18-overexpressing 293T cells were injected subcutaneously into NOD SCID gamma mice. Tumors were analyzed by immunostaining for fibronectin (green), aSMA (red); DAPI, blue. Quantification of percentage fibronectin^{+} tumor area in 293T tumors overexpressing GFP or ADAMTS18 ($p<0.0001$, n=4). (**g**) Lentiviral overexpresion of GFP and ADAMTS18 in mouse myoblasts also overexpressing VEGFA. (**h**) ADAMTS18 does not alter amount of soluble VEGFA. Amount of supernatant VEGFA from myoblasts expressing VEGFA with GFP or ADAMTS18, n=1. (**i**) ADAMTS18 does not alter the ability of VEGFA to signal through VEGFR2. Conditioned medium from VEGFA-expressing myoblasts overexpressing GFP or ADAMTS18 were transferred to starved HUVECs, samples were taken at indicated timepoints and western blots were probed with pVEGFR2 and VEGFR2 antibodies. Representitave of 3 independent experiments. Scale bars: 100mm: f; 50mm: a; 20mm: d. ****$P < 0.0001$ 2-tailed unpaired Student's t test. All values shown as mean ± SD. Source data are provided as a Source Data file.

Supplementary Figure 8

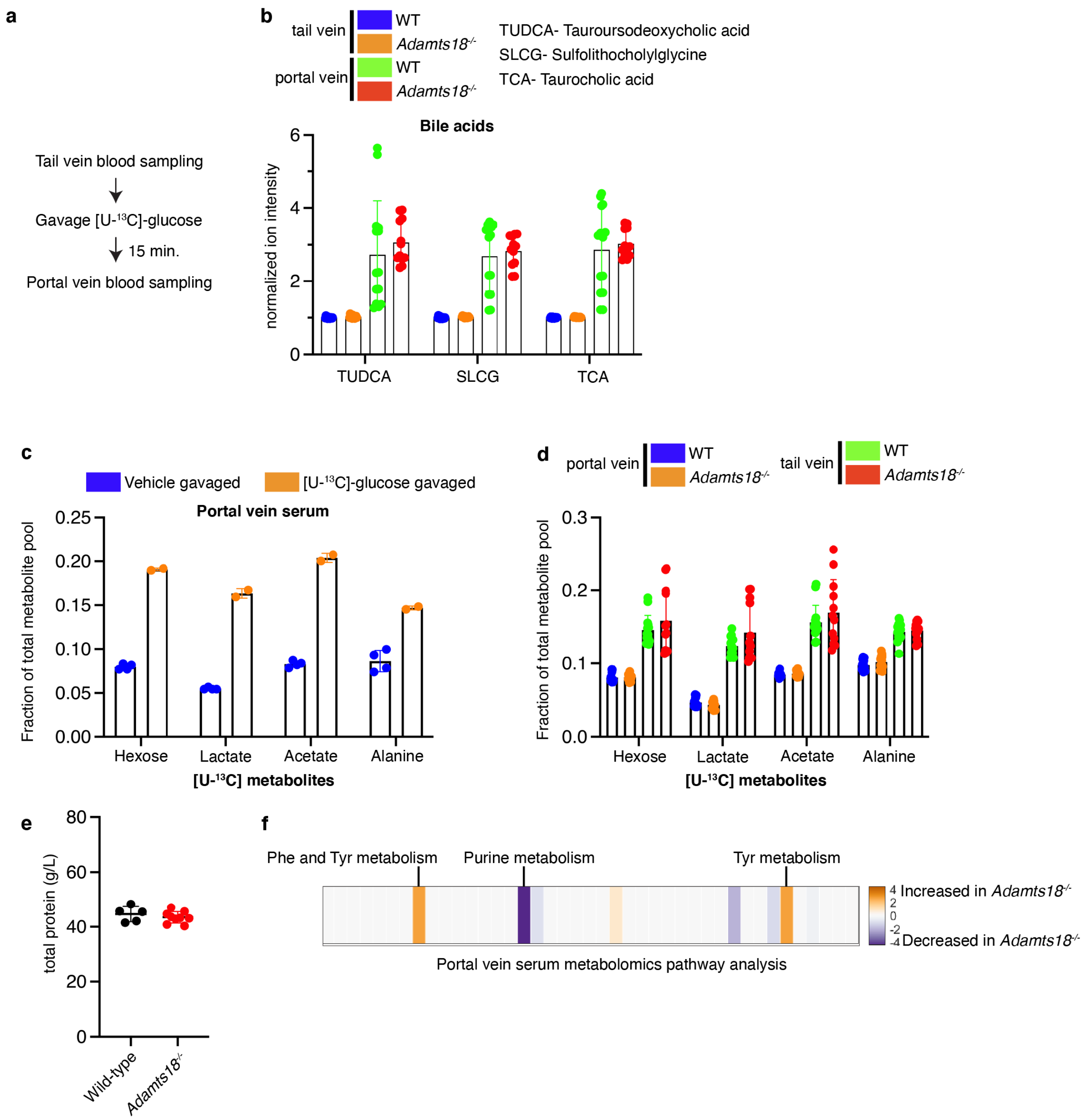

Supplementary Figure 8: Impact of ADAMTS18 deficiency on blood metabolites. (**a**) Scheme of [U-^{13}C]-glucose gavage and blood sampling. (**b, c**) Successful blood sampling strategy determined by (**b**) measurement of bile acids in portal or systemic blood of wild-type and *Adamts18*$^{-/-}$ mice and (**c**) comparison of [U-^{13}C]-glucose and associated metabolites from glucose- and vehicle-gavaged control mice, n=4-5. Note: [U-^{13}C]-labeled metabolites in vehicle-gavaged mice are from naturally occurring ^{13}C isotopes. (**d**) No difference in the amount of portal vein [U-

^{13}C]-labeled glucose between wild-type and *Adamts18*$^{-/-}$ mice, n=4-5. (**e**) No difference in portal venous blood total protein between wild-type and *Adamts18*$^{-/-}$ mice, n=5-9. (**f**) Metabolite pathway analysis of portal vein serum metabolites differentially found between wild-type and *Adamts18*$^{-/-}$ mice, n=5-9. Each band of the horizontal line represents one metabolic pathway, in no specific order. The scale is the -$\log_{10}$ of the adjusted p-value of the enrichment of the metabolites for a particular pathway. The higher the absolute number, the lower the p-value (ie, positive values indicate an increase and negative a decrease in pathway intermediates in portal vein blood from *Adamts18*$^{-/-}$ mice). All values shown as mean ± SD. Source data are provided as Source Data and Supplementary Data files.

Supplementary Figure 9

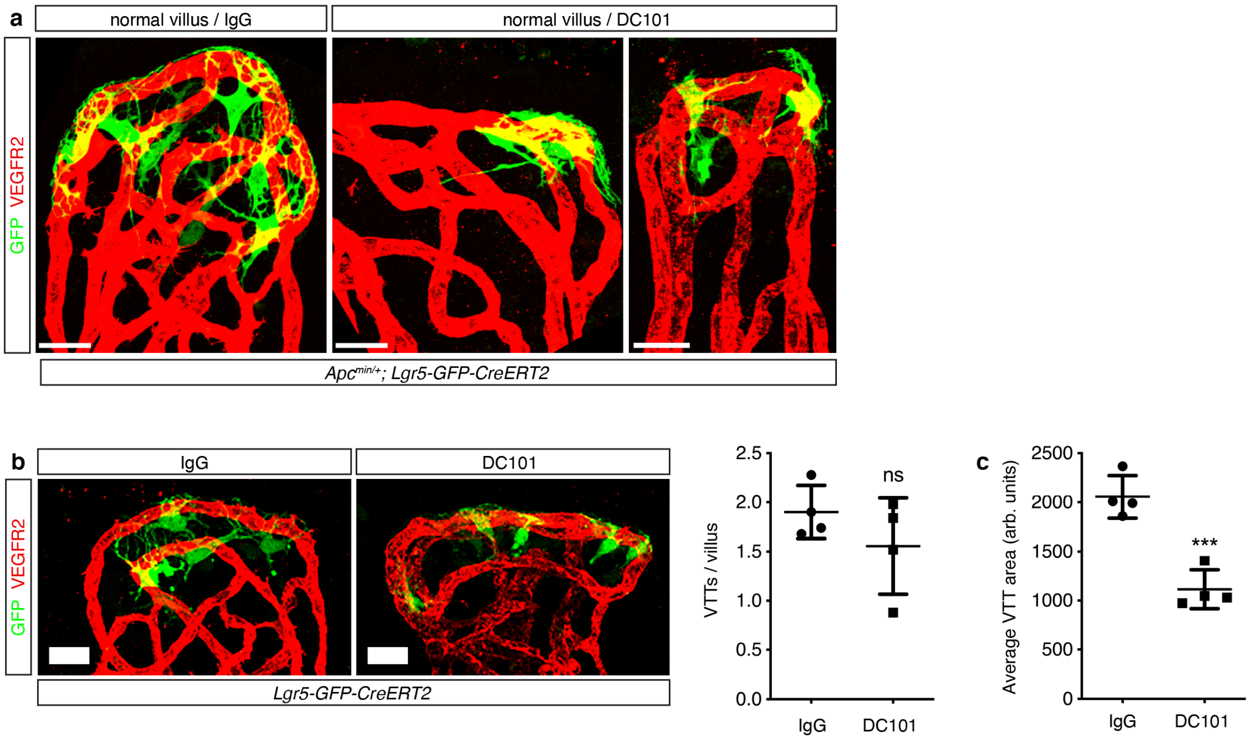

Supplementary Figure 9: VEGFA signaling blockade alters VTT patterning. (**a, b**) DC101 treatment decreases the distance between VTTs (green, GFP) and limits the branching of their stellate extensions near intestinal blood vessels (red, VEGFR2), without decreasing their numbers. Quantification of the number of VTTs/villus tip, n=4. (**c**) VTTs display decreased cellular extensions after VEGFA signaling blockade. Quantification of the average VTT area/villus tip (p=0.0007, n=4). Scale bars: 20mm: a, b. ***P < 0.001 2-tailed unpaired Student's t test. All values shown as mean ± SD. Source data are provided as a Source Data file.

Supplementary Table 1: Antibodies used in this work.

	Antibody (host)	Supplier	Catalog number/clone	Dilution
**Wholemount**	αSMA (mouse)	Sigma-Alrich	C6198 / 1A4	1:750
	Desmin (rabbit)	Millipore	04-585	1:500
	Epcam (rat)	Biolegend	118201 / G8.8	1:200
	ERG (rabbit)	Abcam	ab92513	1:500
	ESM1 (goat)	R&D	AF1999	1:200
	F4/80 (rat)	Invitrogen	MF48000 / BM8	1:200
	fibronectin (rabbit)	Millipore	AB2033	1:200
	GFAP (chicken)	Abcam	ab4674	1:200
	GFP (rabbit)	Abcam	ab290	1:10,000
	GFP (rat)	Biolegend	338002 / FM264G	1:400
	Ki67 (rabbit)	Abcam	ab15580	1:200
	LAMA5 (rabbit)	kind gift, Dr. Lydia Sorokin		1:2000
	MADCAM1 (rat)	Biolegend	120702 / MECA-367	1:400
	NG2 (rabbit)	EMD Millipore	AB5320	1:1000
	Pdgfrα (rat)	eBioscience	14-1401-82 / APA5	1:200
	PECAM1 (rat)	BD Pharmingen	557355 / MEC 13.3	1:400
	Periostin (goat)	R&D	AF2955	1:1000
	PGP9.5 (rabbit)	Dako	Z5116	1:200
	S100 (rabbit)	Thermo Scientific	RB-9018	1:200
	Tenascin C (rat)	R&D	MAB2138 / 578	1:500
	VE-cadherin (goat)	R&D	AF1002	1:200
	VEGFA (goat)	R&D	AF493	1:200
	VEGFR2 (goat)	R&D	AF644	1:100
	VEGFR3 (goat)	R&D	AF743	1:100
**Human Paraffin**	ADAMTS18 (rabbit)	from Dr. Cathrin Brisken's lab		1:5000
**Mouse Paraffin**	αSMA (mouse)	Sigma-Alrich	C6198 / 1A4	1:1000
	ESM1 (goat)	R&D	AF1999	1:200
	F3 (rabbit)	Sino Biological	50413-R001	1:200
	fibronectin (rabbit)	Millipore	AB2033	1:150
	Meca32 (rat)	BD Pharmingen	550563 / MECA-32	1:100
	Pimonidazole (mouse)	Hypoxyprobe	HP1-100Kit / 4.3.11.3	1:300
	Periostin (goat)	R&D	AF2955	1:400
	Tenascin C (rat)	R&D	MAB2138 / 578	1:300
	VEGFR3 (goat)	R&D	AF743	1:100
**Cell sorting**	CD31-PE	eBioscience	12-0311-81	1:1000
	CD45-APC-Cy7	BD Biosciences	561037 / 30-F11	1:1000

	EpCAM-FITC	eBioscience	11-5791-82 / G8.8	1:1000
	gp38-AlexaFluor 647	homemade hybridoma	clone 8.1.1	1:800
**Western blot**	actin (rabbit)	Sigma-Alrich	A2066	1:1000
	GFP (rabbit)	Abcam	ab290	1:3000
	pVEGFR2-Tyr1175 (rabbit)	Cell Signaling	2478S	1:1000
	VEGFR2 (goat)	R&D	AF357	1:1000
